# Altered Heme and Redox Homeostasis Underpin Late-onset Alzheimer's Disease

**DOI:** 10.7150/ijbs.116204

**Published:** 2025-08-22

**Authors:** Adedamola Saidi Soladogun, Chantal Vidal, Maria Del Carmen Chacon Castro, Heng Du, Li Zhang

**Affiliations:** 1 Department of Biological Sciences, University of Texas at Dallas, Richardson, TX 75080, USA.; 2 Department of Pharmacology and Toxicology, University of Kansas, Lawrence, KS, USA.

**Keywords:** Alzheimer's disease, heme homeostasis, iPSC-derived stem cells, late-onset Alzheimer's disease, LOAD, redox homeostasis

## Abstract

Early-onset Alzheimer's disease (EOAD) is associated with highly penetrant mutations in genes such as PSEN2, whereas the strongest genetic risk factor for late-onset Alzheimer's disease (LOAD) is the APOE4 allele. Despite intense research efforts, how neuronal dysfunction is initiated in LOAD cases and how the initiating events for EOAD and LOAD differ remain to be clarified. Using biochemical measurements of energy metabolism, heme and redox homeostasis, in combination with RNA-Sequencing analysis, we characterized biochemical and transcriptome differences in neurons differentiated from human EOAD and LOAD iPSC-derived neural stem cells, relative to their respective control neurons. Strikingly, we found that LOAD neurons, not EOAD neurons, are defective in heme and redox homeostasis. The levels of multiple proteins and enzymes involved in heme synthesis, degradation, and oxidative phosphorylation are preferentially decreased in LOAD neurons, not EOAD neurons. Likewise, heme transport is decreased in LOAD neurons. ROS generation is strongly increased in LOAD neurons, not EOAD neurons. Further, many genes involved in heme and redox homeostasis, as well as cellular energy generation, are downregulated in LOAD neurons, not EOAD neurons. Together, these results strongly suggest that altered heme and redox homeostasis in LOAD neurons underlie the initiation of neurological deficits.

## Introduction

Alzheimer's disease (AD) is characterized by the development of extracellular amyloid beta (Aβ) and neurofibrillary tangles (NFT) in the intracellular environment, neuronal death, and synaptic loss in the brain 1, 2. AD cases can be categorized according to the age of onset: Early-onset Alzheimer's disease (EOAD), which presents in patients younger than 65 years, and late-onset Alzheimer's disease (LOAD), which presents in patients older than 65 years 3. EOAD accounts for 5%-6% of AD cases 4, 5, 6. Among EOAD, 10-15% of the cases show highly penetrant mutations in the amyloid beta precursor protein (APP), presenilin 1 (PSEN1) and presenilin 2 (PSEN2) genes 7, 8. About two-thirds of people diagnosed with LOAD are women 9. The apolipoprotein E gene (APOE) alleles are the strongest genetic risk factor for LOAD 10, 11. *APOE* is the main apolipoprotein present in the brain. It is involved in the transport of lipids and is responsible for their internalization via specific binding to cell-surface lipoprotein receptors 12. Three APOE alleles exist. They differ by nucleotides at two sites in the gene: E2 is considered protective (the rarest allele, 5%-10%), E3 is considered neutral (the most common allele, ~80%), and E4 increases LOAD risk (10%-15%) 13. The AD risk is dose dependent and is modulated by global and local genetic ancestries, other genetic risk loci and the lifetime exposome of an individual 14.

EOAD and LOAD exhibit numerous differences, although they display the same pathological process 3. For example, the decrease in functional connectivity from the hippocampus to other brain regions is different between patients with EOAD and LOAD 15, and EOAD and LOAD show distinct cognitive features 16. Interestingly, APOE4 carriers exhibit slower cognitive decline in general cognitive function, language, memory, and frontal-executive function than non-carriers in EOAD, but not in LOAD 17. APOE4 carriers in LOAD have younger symptom onset, but APOE E4 carriers in EOAD had older symptom onset 18. Likewise, APOE4 was shown to cause differential effects on neurological clinical phenotypes in LOAD and EOAD patients 19. Further, the same APOE4 mutation exerts differential effects on the onset of EOAD and LOAD. Increasingly larger genome-wide association studies (GWAS) and meta-analysis have provided important insights into the genetics of AD 20-23, and have identified 101 independent AD-associated single nucleotide polymorphisms across 81 genome-wide significant loci 24. These lines of evidence show that APOE4 interacts with many other factors or genes to initiate the genesis of neuronal deficits associated with LOAD. Indeed, APOE4 causes very different neurological outcomes in different patients 17-19. Thus, studies involving isogenic iPSC-derived neural stem cells expressing different alleles of APOE is unlikely to uncover the full spectrum of molecular events leading to the etiopathogenesis of LOAD.

Additionally, iPSC cells, as pluripotent stem cells, exhibit the properties of fetal-stage cells and are indistinguishable from ESCs in the expression of age-related markers; this iPSC rejuvenation is known to occur at the level of epigenetic methylated DNA, transcriptome, and functional phenotypes, such as mitochondrial function, cellular senescence, and DNA damage (for comprehensive reviews, see refs. 25, 26). Even somatic cells from centenarian and supercentenarian donors can be reprogrammed to iPSC cells that are indistinguishable from hESCs 27, 28. iPSC cells enable the modeling of susceptibility rather than normal disease or disease progression. Directly converted neurons (iNs) have been used to model age-related neurodegenerative diseases recently 29. Nonetheless, the ESCs-like properties of iPSC cells allow us to examine cellular events that are linked to only genetic features in donors' somatic cells, from which iPSCs are derived, without the complication of age and environmental factors. While this study cannot gain insights into the role of immune systems, it can reveal these molecular and cellular changes existed in fetal neurons of AD patients. Such analysis has the potential to identify new pathological mechanisms that may be masked in studies of aged patients due to the complication of aging and the environment.

In this report, we took advantage of neurons differentiated from iPSC-derived neural stem cells generated from two scarcely available pairs of sex-matched cell lines representing healthy control neurons and EOAD/LOAD neurons. Specifically, we examined how heme homeostasis and OXPHOS are affected in EOAD and LOAD neurons. Multiple subunits in OXPHOS complexes II-IV contain heme 30. As neurons differentiate, higher levels of heme are required to support OXPHOS 31. Mutations or alterations that disturb heme homeostasis can cause neurological dysfunction 32. Here, our data strongly suggest that the initiating molecular events underlying EOAD and LOAD are distinct, with altered heme homeostasis and oxidative metabolism being a determining factor in LOAD etiopathogenesis.

## Materials and Methods

### iPSC Cell Culture and Differentiation

iPSC-derived neural stem cells from four different donors (Table 1) were purchased from Axol Bioscience and cultured according to the manufacturer's guidelines. No personal identifying information of patients was revealed. These two pairs of iPSC cell lines derived from EOAD and LOAD patients and healthy controls were found to be best matched among available lines in commercial vendors and repositories. Cells were maintained in a 5% CO_2_ atmosphere at 37°C in tissue culture-grade 6-well plates coated with Surebond (Axol Bioscience). The culture medium, Neural Maintenance Media (Axol Bioscience) supplemented with EGF (20 ng/ml) and FGF2 (20 ng/ml), was refreshed every two days until the cells reached 70% confluence. Next, cells were detached using Unlock (Axol Bioscience) and re-plated at a density of 70,000 cells/cm². For details, see https://axolbio.com/user-protocols/neuroscience-user-protocols/.

For neuronal differentiation, iPSC-derived neural stem cells were seeded onto Surebond XF (Axol) pre-coated plates using Neural Plating Media (Axol Bioscience). After 24 hours, the medium was changed to Neural Maintenance Media without EGF and FGF2. After an additional 24 hours, the cells were switched to Neural Differentiation-XF Media, with media changes occurring every three days. On the sixth day, half of the medium was replaced with Neural Maintenance-XF Media (Axol Bioscience). Another 24 hours later, half of the medium was again replaced with fresh Neural Maintenance-XF Media. The cells were cultured for a total of 35 days, with media changes every three days.

For assays involving undifferentiated cells, measurements were carried out prior to the initiation of neuronal differentiation process. For assays involving differentiated neurons (DIF), measurements were carried out prior after the 35-day differentiation period. Five experimental conditions were established and compared: undifferentiated iPSC-derived neural stem cells from healthy controls (healthy control, HC M/F UD), differentiated healthy control neurons (HC M/F DIF), differentiated healthy control neurons treated with Aβ_42_ treatment (HC M/F DIF AB), undifferentiated iPSC-derived neural stem cells from AD patients (AD PSEN1/APOE4 UD), and differentiated AD neurons (AD PSEN1/APOE4 DIF).

### Oligomeric Aβ_42_ preparation

Oligomeric Aβ_42_ was prepared following established procedures 33. Aβ1-42 peptide (GenicBio) was dissolved in 1,1,1,3,3,3-hexafluoro-2-propanol (HFIP) to achieve a concentration of 1 mM, utilizing glass gas-tight Hamilton syringes with Teflon plungers. This solution was then divided into aliquots in micro centrifuge tubes and allowed to evaporate in a fume hood until dry. The dried peptide film was reconstituted in DMSO to a concentration of 5 mM and sonicated for 10 minutes in a bath sonicator. The peptide solution was then diluted with cold Neural Maintenance-XF Media to a final concentration of 100 µM and vortexed for 30 seconds. This solution was incubated at 4 °C for 24 hours to facilitate oligomer formation. The oligomeric Aβ (0.5 µM) was then applied to neurons that had been differentiated for 35 days and incubated for 24 hours before assays.

### Immunocytochemistry (ICC)

Immunocytochemistry was performed on neurons plated in 96-well plates following the established protocol 34. For proteins localized in mitochondria, 100 µL of Mitotracker Red (Thermo Fisher Scientific), diluted in Neural Maintenance-XF Media to a final concentration of 100 nM, was added to each well. The cells were then incubated at 37°C for 45 minutes. The cells were fixed with 4% formaldehyde (75 µL/well) for 20 minutes and rinsed three times (100 µL/well) for 5 minutes with a blocking buffer containing 2 mg/mL BSA in PBS. Next, the cells were blocked and permeabilized in 2 mg/mL BSA in PBS with 0.2% Triton X-100 for 30 minutes in a humidity chamber at room temperature. Following this, the cells were rinsed three times for 5 minutes each with 2 mg/mL BSA in PBS. Primary antibodies were diluted in PBS containing 2 mg/mL BSA and were added to corresponding wells. The primary antibodies used were: Anti-ALAS1 (Abcam catalog no. ab154860), anti-NF M (SYSY antibodies, catalog no. 171231), Anti-FECH (Santa Cruz Biotechnology catalog no. sc-99138), Anti-HO-1 (Novus Biologicals catalog no. NBP1-97507), Anti-HO-2 (Abcam catalog no. ab90515), Anti-BLVRB (Novus Biologicals catalog no. NBP1-83435), Anti-PDHA1 (1:100, Abcam catalog no. ab92696), Anti-APP (1:100, Abcam catalog no. ab15272), Anti-4HNE (1:100, Abcam catalog no. ab46545), Anti-COX5B (1:500, Abcam catalog no. ab180136), Anti-MTCO2 (1:100, Abcam catalog no. ab79393), and Anti- SLC48A1 (1:100, catalog no. NBP1-91563).

Cells were incubated with the primary antibodies overnight in a humidity chamber. Next, cells were rinsed three times for 5 minutes each with 2 mg/mL BSA in PBS. Cells were then incubated with the corresponding secondary antibodies, Alexa Fluor 488 (Thermo Fisher Scientific, 1:100) or Alexa Fluor 594 (Thermo Fisher Scientific, 1:100), diluted in 2 mg/mL BSA in PBS, for 1 hour at room temperature in a humidity chamber in the dark. Following this incubation, the cells were rinsed four times for 5 minutes each with 2 mg/mL BSA in PBS. For nuclear staining, cells were incubated with DAPI diluted in PBS (100 µL, 1:1000) for 5 minutes and then rinsed with 100 µL of PBS. After the final wash, 50 µL of Vectashield mounting medium was added to the cells, and cells were imaged. Imaging was conducted using a Biotek Cytation 5 plate reader (DAPI 377/447; GFP 469/525; Texas Red 586/647). Fluorescence intensity was quantified using ImageJ. At least 5 different regions of interest (ROI) of proteins localized with markers were selected, and the integrated density was measured. ROIs were also selected for the background signals. The relative fluorescence unit (RFU) was calculated as:

RFU = (Integrated Density of ROI - Integrated Density of Background)/Area of ROI

### Measurements of heme uptake and heme export

Heme uptake was measured using the fluorescent heme analogs, zinc (II) protoporphyrin IX (ZnPP, Frontier Scientific, catalog no. Zn 625-9) as previously described 35. Briefly, 20,000 induced pluripotent stem cell (iPSC)-derived neuronal cells were seeded in 96-well plates and incubated with 60 μL of either ZnPP in complete media for 3 hours at 37°C. Fluorescence intensity was measured using a Biotek Cytation 5 plate reader with excitation at 425 nm and emission at 594 nm. Background fluorescence from wells without ZnPP was subtracted. Measurements were performed in multiple replicates, and ZnPP uptake was normalized to total cell count. Heme export was measured following the established protocol for heme uptake, with modifications to measure efflux. After the initial 3-hour incubation with ZnPP, the medium was carefully removed and replaced with fresh complete media. Cells were then incubated for an additional 3 hours at 37°C. Fluorescence intensity was measured again using the Biotek Cytation 5 plate reader (excitation 425 nm, emission 594 nm). Heme export was calculated as the difference between the fluorescence intensity after the initial 3-hour incubation and the fluorescence intensity after the subsequent 3-hour incubation in fresh medium, after correcting for background fluorescence from wells without the analogs. The resulting value was normalized to the total cell number.

### Measurement of reactive oxygen species (ROS)

Reactive oxygen species (ROS) levels were quantified using the DCFDA/H2DCFDA-Cellular ROS Assay Kit (ab113851, Abcam) following the manufacturer's protocol for adherent cells, with minor modifications. Briefly, cells were seeded at a density of 20,000 cells/cm² in a 96-well plate. The cells were then washed with 1X Buffer and incubated with the diluted DCFDA solution for 45 minutes at 37°C in the dark. Following incubation, the DCFDA solution was removed, and cells were washed again with 1X Buffer. Fluorescence intensity was immediately measured using a Biotek Cytation 5 plate reader (excitation 485 nm, emission 535 nm). Background fluorescence from wells containing cells without DCFDA was subtracted to account for any non-specific signal.

### Measurements of NAD and NADH levels

The NAD and NADH levels were measured using the NAD+/NADH-Glo Assay Kit (G9071, Promega) according to manufacturer's instructions. Briefly, neuronal cells were seeded at a density of 20,000 cells/cm² in a 96-well black plate with a clear bottom and allowed to adhere overnight. The cells were then washed with 1X Buffer and incubated with 50 µL of base solution containing 1% DTAB to ensure cell lysis. After mixing briefly on a plate shaker, 50 µL of each sample was transferred to an empty well for acid treatment. Next, 25 µL of 0.4N HCl was added, creating the acid-treated samples. The original sample wells were kept as base-treated samples without the addition of HCl. The plate was then covered and incubated at 60°C for 15 minutes. Following incubation, the plate was equilibrated at room temperature for 10 minutes. To neutralize the acid in the acid-treated samples, 25 µL of 0.5M Trizma® base was added to each well. For the base-treated samples, 50 µL of HCl/Trizma® solution was added to each well. At this point, the total volume in each well was 100 µL. An equal volume of NAD/NADH-Glo™ Detection Reagent (100 µL) was then added to each well. The plate was gently shaken to mix the contents, followed by incubation for 60 minutes at room temperature. Luminescence was recorded using a BioTek Cytation 5 cell imaging multimode reader.

### RNA Sequencing (RNA-Seq) analysis

Neural stem cells from late-onset Alzheimer's disease (LOAD) patients and healthy controls were cultured in 6-well plates. Cells were collected at appropriate differentiation stages or after treatment with amyloid beta (Aβ). The collection involved scraping the cells, followed by centrifugation to remove PBS, and storing the samples at -80°C for subsequent RNA sequencing analysis. Total RNA was extracted using the SPLIT Total RNA Kit (Lexogen) following the manufacturer's protocol. RNA integrity was assessed using an Agilent 2100 Bioanalyzer (Agilent Technologies) with RNA integrity numbers (RIN) greater than 7 considered acceptable. RNA-seq libraries were prepared using the QuantSeq 3' mRNA-Seq Library Prep Kit (Lexogen) according to the manufacturer's instructions. Briefly, 40 ng of total RNA was used for library preparation, which included poly-A selection, cDNA synthesis, and library amplification with indexed primers. The libraries were sequenced on an Illumina NextSeq 2000 platform. The average input read lengths range from 87-93. Sequencing yielded an average of approximately 5 million reads per sample. The quality of raw sequencing data was assessed using FastQC (https://www.bioinformatics.babraham.ac.uk/projects/fastqc/). Adapter sequences and low-quality bases were trimmed using CUTADAPT.

Reads were aligned to the human reference genome (GRCh38) using STAR. The average mapping rate was approximately 85-90%. Differential gene expression analysis was conducted with DESeq2. Only genes with an adjusted p-value < 0.05 and log fold change >0.8 or <-0.8 were selected as differentially expressed. GO analysis was carried out using data from HGNC, https://string-db.org/, https://david.ncifcrf.gov/, and https://www.informatics.jax.org/go. RNA-Seq data are available at GEO database: accession number GSE288917.

### Statistical analyses of data

Analysis of Data from ICC and measurements of heme uptake, export, ROS, NAD, and NADH levels was performed with ANOVA. A *P* value of <0.05 was considered statistically significant. Significance levels were indicated by symbols: *, *P* < 0.05, **, *P* < 0.01, ***, *P* < 0.001, ns (not significant), *P* >0.05.

## Results

### Heme homeostasis is differentially affected in EOAD versus LOAD neurons

Our previous studies have shown that the expression of heme synthetis enzymes such as ALAS1 and heme degradation enzyme like HO-2 is significantly reduced in human AD brains compared to control brains 31, 36. However, these detected postmortem changes in AD brains have accumulated high levels of Aβ, which can reduce the expression of heme synthetic and degradation enzymes. Importantly, EOAD/LOAD neurons differentiated from iPSC-derived neural stem cells have not been exposed to high levels of extracellular Aβ. Thus, changes detected in these neurons cannot be attributed to the mere exposure to Aβ. Rather, such changes may represent early events in AD etiopathogenesis, as suggested by a comprehensive review of experimental data on APOE4 37. To probe such crucial early events, we acquired two scarcely available pairs of iPSC-derived neural stem cell lines (Axol Bioscience) generated from human dermal fibroblasts. The EOAD and LOAD cell lines were generated from cells of a male donor carrying an M146L mutation in the PSEN1 gene and from cells of a female donor carrying a homozygous APOE4 allele, respectively (Table 1). The control cell lines were matched with the AD lines in sex and APOE status to the extent possible, for optimal detection of the effects of genetic mutations linked to EOAD and LOAD patients. The matched EOAD pair shares a common E2 allele, while the matched LOAD pair provides a clear comparison of E3/E3 and E4/E4 (Table 1).

**Figure 1:**
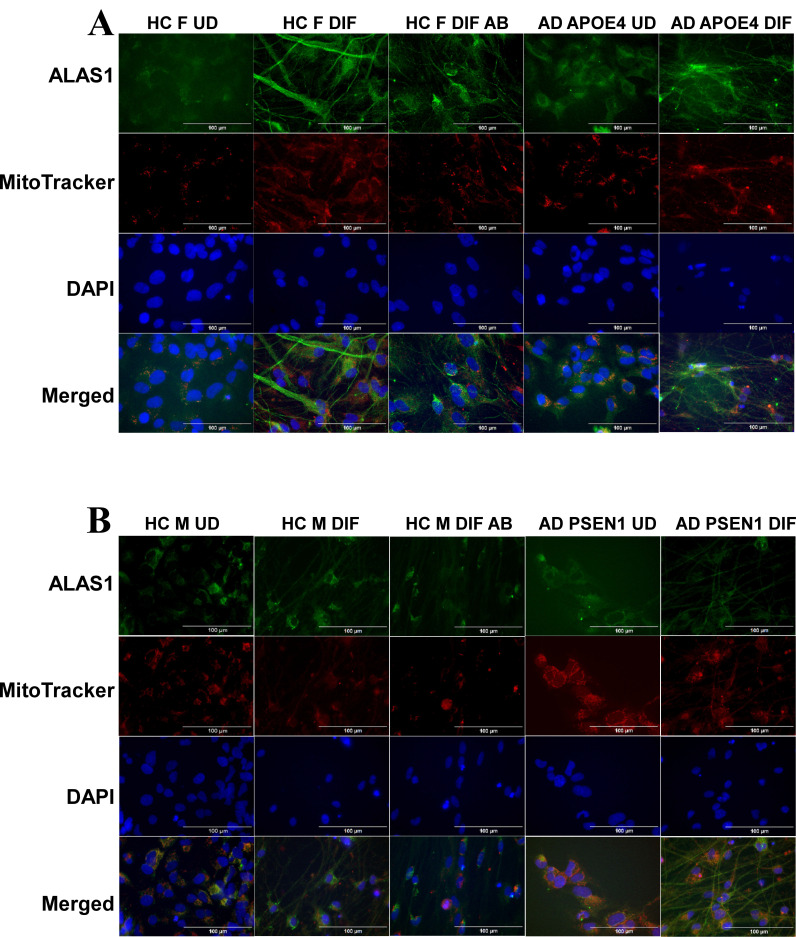
Immunocytochemistry images showing ALAS1 levels in undifferentiated (UD) neural stem cells and differentiated (DIF) neurons derived from iPSC neural stem cells. (A) Representative fluorescent images of undifferentiated (UD) and differentiated (DIF) male (M) healthy control (HC) and early-onset Alzheimer's disease (EOAD, PSEN1 mutation) neural stem cells. Cells were stained with anti-ALAS1 (5-aminolevulinate synthase) antibody (green), MitoTracker (red), and DAPI (blue). The merged images indicate the overlap of ALAS1 with MitoTracker, demonstrating the mitochondrial localization of ALAS1. HC M UD, HC M DIF, HC M DIF AB, EOAD UD, and EOAD DIF conditions are shown. (B) Representative fluorescent images of undifferentiated (UD) and differentiated (DIF) female (F) healthy control (HC) and late-onset Alzheimer's disease (LOAD, APOE4 mutation) neural stem cells. Similar to panel A, cells were stained with anti-ALAS1 antibody (green), MitoTracker (red), and DAPI (blue). Merged images confirm the mitochondrial localization of ALAS1. HC FUD, HC F DIF, HC F DIF AB, LOAD UD, and LOAD DIF conditions are displayed. Scale bars:100 μm.

**Figure 2:**
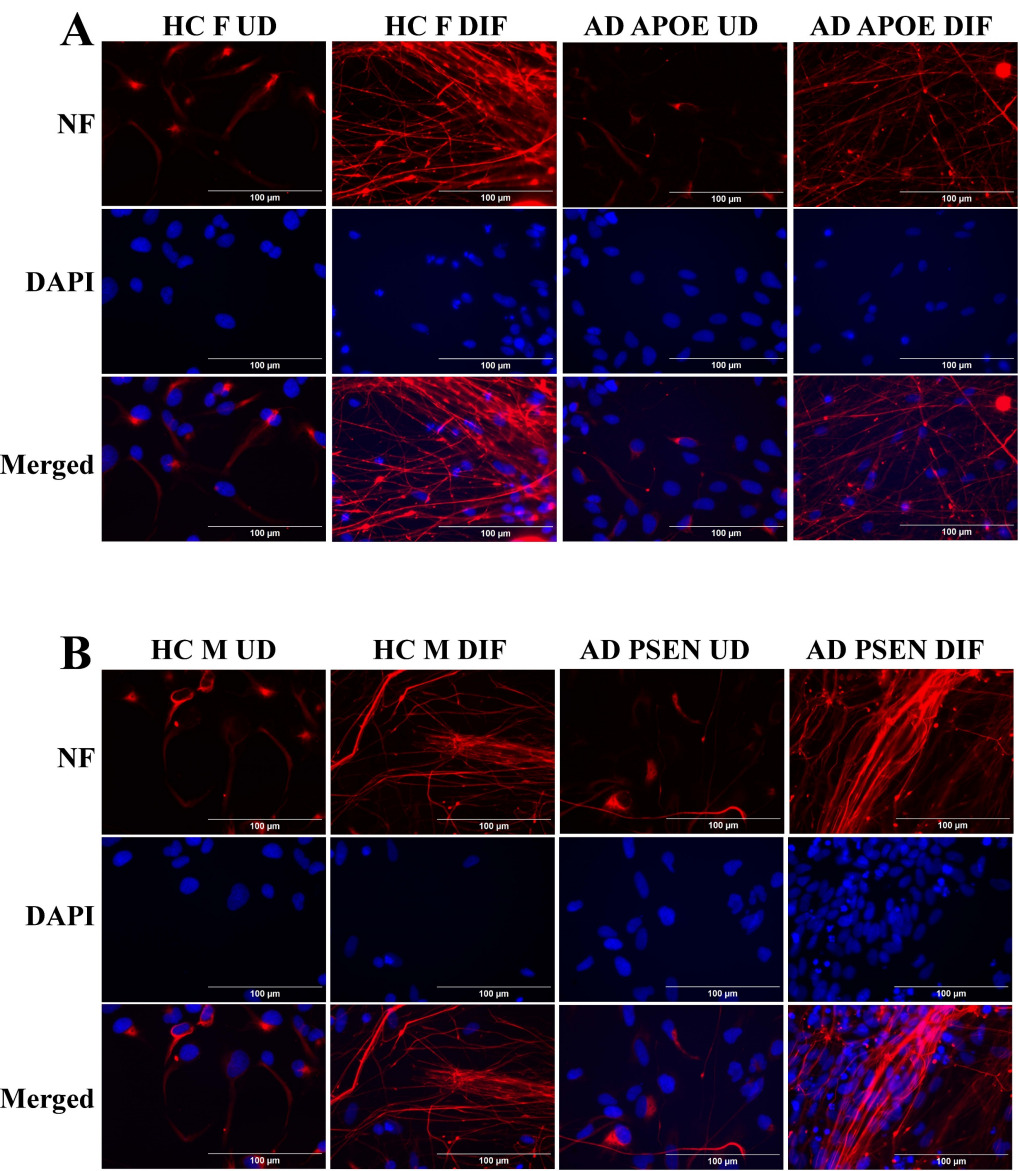
Immunocytochemistry images showing neurofilament protein NF-M in differentiated neurons derived from iPSC neural stem cells. (A) Representative fluorescent images of differentiated neurons from healthy control (HC) and early-onset Alzheimer's disease (EOAD, PSEN1 mutation) iPSC cells. (B) Representative fluorescent images of differentiated neurons from healthy control (HC) and late-onset Alzheimer's disease (LOAD, APOE4 mutation) iPSC cells. Scale bars:100 μm.

**Figure 3:**
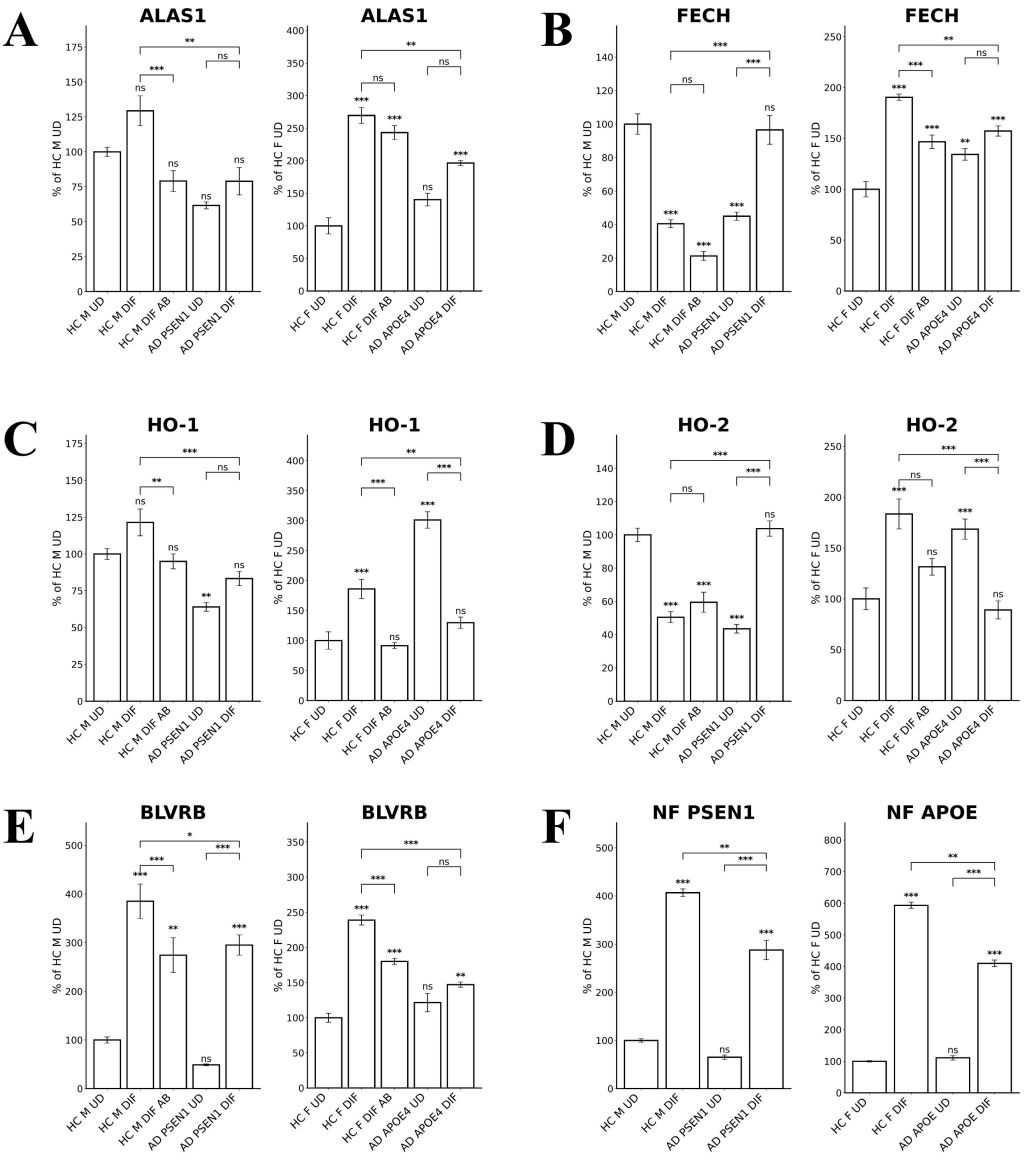
Quantified levels of heme synthesis enzymes ALAS1 and FECH as well as heme degradation enzymes HO-1, HO-2, and BLVRB reveal elevated levels of FECH and HO-2 in EOAD neurons but reduced levels of FECH and HO-2 in LOAD neurons. (A) Normalized relative fluorescence units (RFU) indicating levels of ALAS1 in undifferentiated (UD) and differentiated (DIF) male (M) healthy control (HC) neurons, as well as differentiated neurons treated with Aβ (HC M DIF AB), early-onset Alzheimer's disease (EOAD, AD PSEN1 UD, AD PSEN1 DIF), and late-onset Alzheimer's disease (LOAD, AD APOE4 UD, AD APOE4 DIF). (B) Normalized RFU indicating levels of FECH. The cells and treatment conditions in B-E are labeled in the same way as in (A). (C) Normalized RFU indicating levels of HO-1. (D) Normalized RFU indicating levels of HO-2. (E) Normalized RFU indicating levels of BLVRB. (F) Normalized RFU indicating levels of NF. Data are presented as mean ± standard error of the mean (SEM). N = 5. Statistical significance: *, P < 0.05, **, P < 0.01, ***, P < 0.001, ns: not significant. Indicated P values directly above the bars were calculated by comparing the indicated cells with undifferentiated healthy control cells.

To detect the levels of proteins/enzymes associated with heme synthesis, degradation, and transport, we carried out immunocytochemistry in undifferentiated neural stem cells (both control and AD), differentiated neurons (both control and AD), and in differentiated neurons treated with Aβ (for healthy control only). For example, Figure 1A & B show the fluorescent images of these cells stained with anti-ALAS1 (5-aminolevulinate synthase) antibody, DAPI, and MitoTracker. The ALAS1 staining pattern overlaps with that of MitoTracker, indicating the mitochondrial localization of ALAS1. Figure 2A & B show the fluorescent images of these cells stained with anti-NF (neuronal marker neurofilament protein NF-M) antibody. Quantification of the signals allows comparison of the levels of proteins/enzymes between AD and control neurons. As expected 31, Aβ reduced the levels of ALAS1 (Figure 3A) and ferrochelatase FECH (Figure 3B) in both control neurons. ALAS1 levels were significantly reduced in EOAD neurons carrying the PSEN1 mutation (AD PSEN1 DIF, Figure 3A) and LOAD neurons carrying APOE4 allele (AD APOE4 DIF, Figure 3A), compared to their respective control neurons (HC M DIF & HC F DIF, Figure 3A). Interestingly, FECH levels were increased in EOAD neurons (AD PSEN1 DIF, Figure 3B) but decreased in LOAD neurons (AD APOE4 DIF, Figure 3B). Next, we detected the levels of enzymes involved in heme degradation, including heme oxygenases HO-1 and HO-2, as well as biliverdin reductase BVRB. HO-1 levels were reduced in both EOAD (AD PSEN1 DIF, Figure 3C) and LOAD neurons (AD APOE4 DIF, Figure 3C), compared to their respective control neurons (HC M DIF & HC F DIF, Figure 3C). However, HO-2 levels (Figure 3D), like FECH levels, were increased in EOAD neurons but reduced in LOAD neurons. BLVRB levels were reduced in both EOAD and LOAD neurons (Figure 3E). The levels of neurofilament NF-M were somewhat increased in both EOAD and LOAD neurons relative to their controls, consistent with a previous study showing that NF-M is increased in AD iPSC-derived cultures 38.

**Figure 4:**
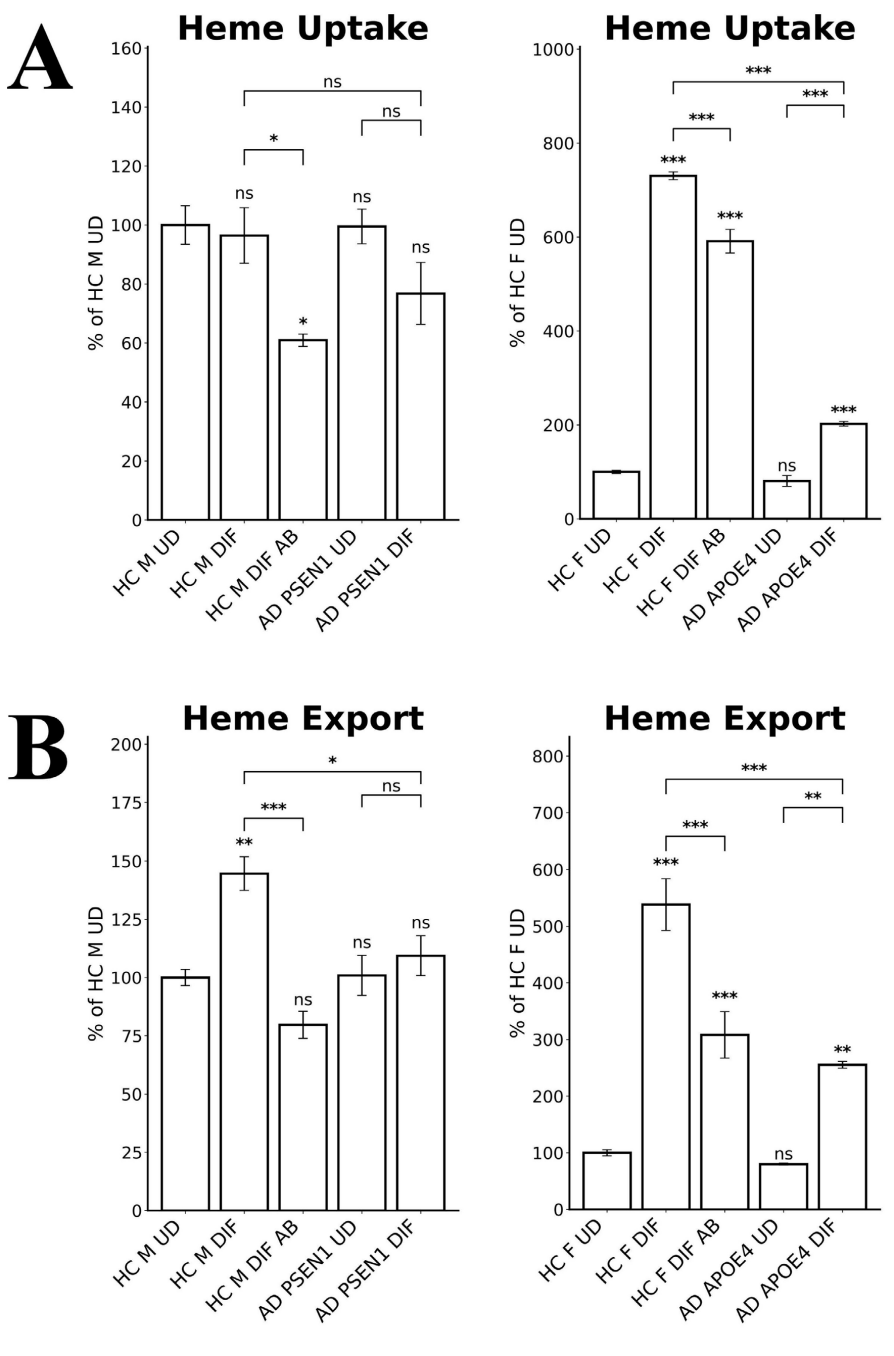
The levels of heme uptake are differentially affected in LOAD vs EOPAD neurons. (A) Measurements of heme uptake reveal reduction of heme uptake in LOAD neurons, but no significant change in EOAD neurons. (B) Measurements of heme export reveal decreases in EOAD and LOAD neurons. Data are presented as mean ± standard error of the mean (SEM). N = 4. Statistical significance: *, P < 0.05, **, P < 0.01, ***, P < 0.001, ns: not significant. Indicated P values directly above the bars were calculated by comparing the indicated cells with undifferentiated healthy control cells.

Heme transport plays important roles in neuronal functions. We therefore detected the levels of heme uptake and export in EOAD and LOAD neurons. We measured heme uptake and export, using zinc protoporphyrin IX (ZnPP), a fluorescent heme analogue, as described previously 35. Due to its strong fluorescence, ZnPP/ZnMP has been used to qualitatively detect heme uptake and to make relative comparison of heme uptake and export in organisms and cells ranging from fungi to mammals 39-41. The authors' lab has previously shown that heme and ZnPP compete with each other in their import to various cell lines 35. Indeed, Figure 4A shows that heme uptake in EOAD neurons (AD PSEN1 DIF) was largely unaffected whereas heme uptake in LOAD neurons (AD APOE4 DIF) was strongly reduced, compared to respective control neurons (HC M DIF & HC F DIF). Figure 4B shows that heme export was slightly reduced in EOAD (AD PSEN1 DIF) neurons and moderately reduced in LOAD (AD APOE4 DIF) neurons. Similarly, Aβ reduced heme uptake and export more strongly in LOAD neurons than in EOAD neurons (Figure 4A & B).

All these results together suggest a clear difference in heme homeostasis between EOAD and LOAD neurons: In LOAD (AD APOE4 DIF) neurons, detected enzymes/proteins involved in heme synthesis (ALAS1, Figure 3A; FECH, Figure 3B) and degradation (HO-1, Figure 3C; HO-2, Figure 3D; BLVRB, Figure 3E) were all significantly reduced relative to control neurons. Further, heme uptake (Figure 4A) was also strongly reduced in LOAD (AD APOE4 DIF) neurons relative to control neurons (HC F DIF). In contrast, in EOAD neurons (AD PSEN1 DIF), heme synthetic enzyme FECH (Figure 3B), and heme degradation enzyme HO-2 (Figure 3D) were actually increased. Such increases are likely to compensate for the reduction of other related enzymes in EOAD neurons. Heme uptake in EOAD neurons (Figure 4A) was largely unaffected. These results show that heme synthesis, degradation, and uptake are decreased in LOAD neurons, but not EOAD neurons, indicating preferentially impaired heme homeostasis in LOAD neurons.

### Mitochondrial respiratory complex subunits and NADH levels were more strongly reduced in LOAD versus EOAD neurons

**Figure 5:**
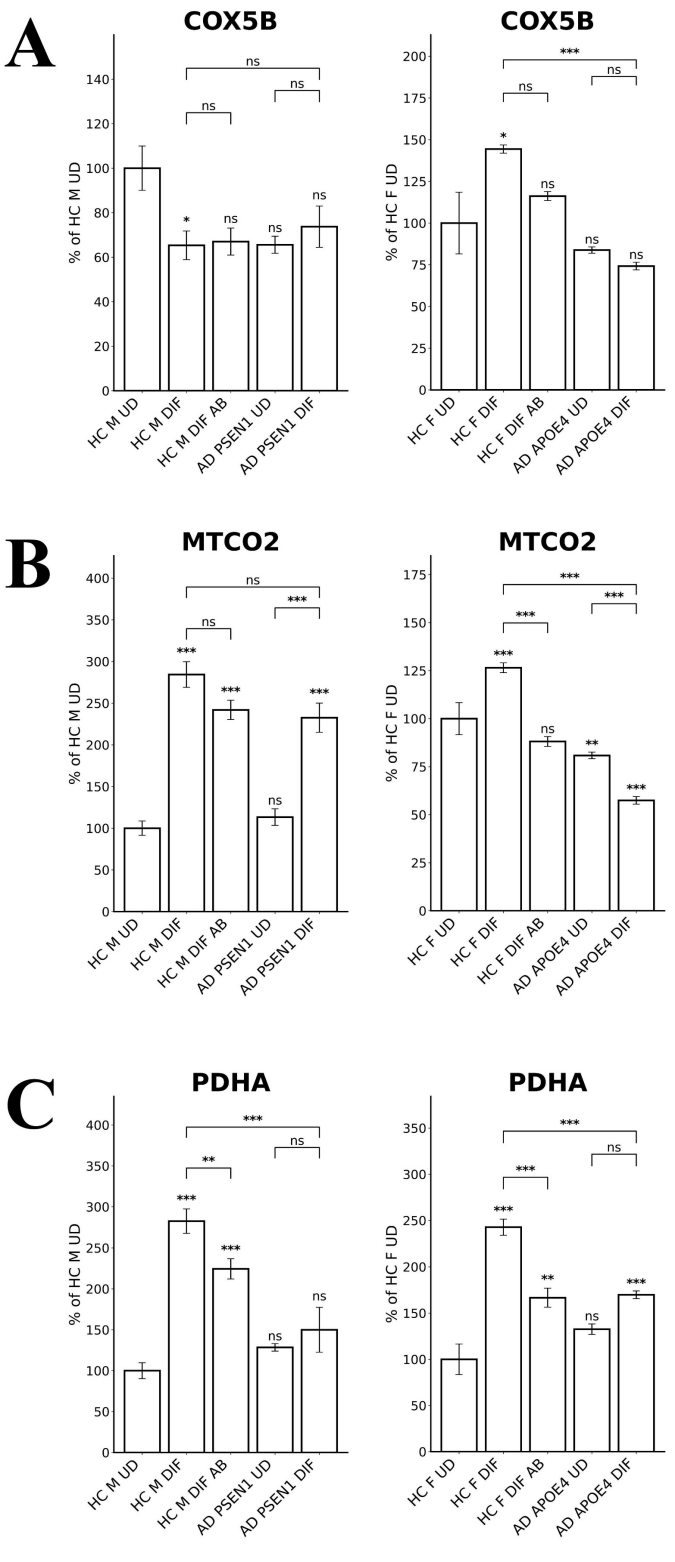
The levels of enzymes involved in oxidative metabolism are differentially affected in LOAD vs EOPAD neurons. (A) Quantified levels of subunits of cytochrome c oxidase COX5B. (B) Quantified levels of subunits of cytochrome c oxidase MTCO2. (C) Quantified levels of the subunit of pyruvate dehydrogenase complex PDHA. Data are presented as mean ± standard error of the mean (SEM). N = 5. Statistical significance: **, P < 0.01, ***, P < 0.001, ns: not significant. Indicated P values directly above the bars were calculated by comparing the indicated cells with undifferentiated healthy control cells.

To probe the differences in oxidative phosphorylation (OXPHOS), we measured the levels of several OXPHOS complex subunits in LOAD versus EOAD neurons. Figure 5A shows that COX5B (Cytochrome C Oxidase Subunit 5B) levels were unaffected in EOAD neurons but significantly reduced in LOAD neurons. Likewise, the levels of MTCO2 (Mitochondrially Encoded Cytochrome C Oxidase II) were decreased by less than 30% in EOAD neurons but were strongly reduced in LOAD neurons (Figure 5B). The levels of the TCA enzyme subunit PDHA (Pyruvate Dehydrogenase E1 Subunit Alpha 1) were reduced in both EOAD and LOAD neurons (Figure 5C). Consistent with previous studies showing that APOE4 reduces OXPHOS activity 42, 43, these results suggest that OXPHOS is more severely affected in LOAD neurons than in EOAD neurons.

**Figure 6:**
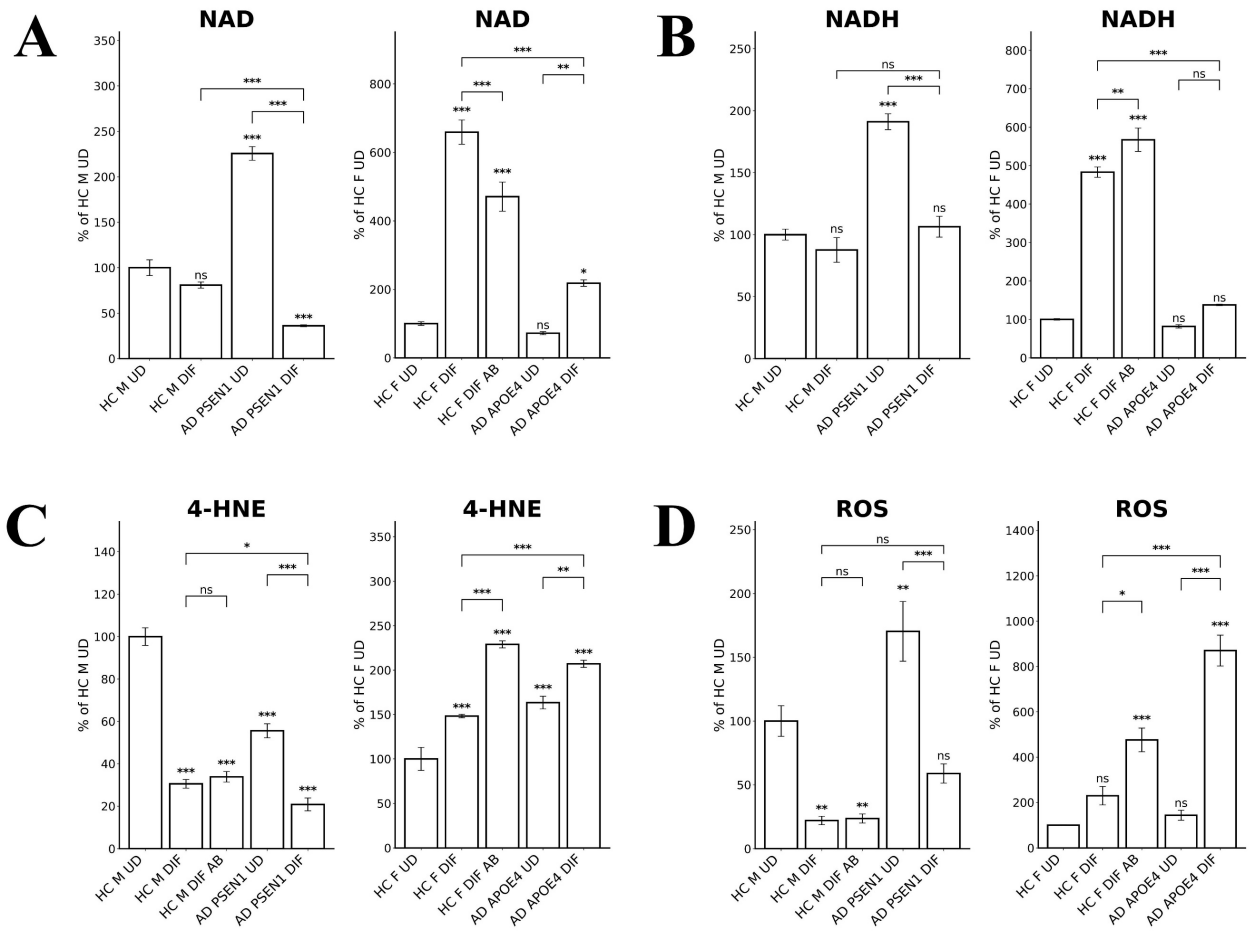
Measurements of NAD, NADH, and ROS levels reveal greater imbalance in LOAD neurons compared to EOAD neurons. (A) NAD levels were reduced in both EOAD and LOAD neurons, with LOAD neurons showing a more pronounced reduction. (B) NADH levels were unchanged in EOAD neurons but were strongly decreased in LOAD neurons. (C) 4-HNE levels, a marker of lipid peroxidation, were increased in LOAD neurons but decreased in EOAD neurons. Aβ treatment increased 4-HNE levels in healthy control neurons. (D) ROS levels were significantly elevated in LOAD neurons, but not changed in EOAD neurons. Aβ treatment also resulted in a significant increase in ROS levels in healthy control neurons. Data are presented as mean ± standard error of the mean (SEM). N = 4. Statistical significance: *, P < 0.05, **, P < 0.01, ***, P < 0.001, ns: not significant. Indicated P values directly above the bars were calculated by comparing the indicated cells with undifferentiated healthy control cells.

NAD and NADH play important roles in energy metabolism, and reduced NAD/NADH levels have been shown to be associated with aging and AD 44-47. Particularly, NADH is used to generate ATP via OXPHOS and for generating the key intracellular antioxidant glutathione (GSH). Thus, we directly measured the levels of NAD and NADH. Figure 6A shows that NAD levels were both reduced in EOAD and LOAD neurons, although the degree of reduction was more severe in LOAD neurons. However, NADH levels were somewhat increased in EOAD neurons, but strongly decreased in LOAD neurons (Figure 6B). Together, these results strongly suggest that energy metabolism and NADH levels are more severely deficient in LOAD neurons versus EOAD neurons.

### Redox homeostasis was more severely affected in LOAD neurons compared to EOAD neurons

To further confirm the status of redox homeostasis in LOAD and EOAD neurons, we detected the levels of lipid peroxidation, using antibodies to 4-Hydroxynonenal. We found that lipid oxidation was decreased in EOAD neurons but increased in LOAD neurons (Figure 6C). Measurement of ROS levels showed that the increase in ROS levels was much more prominent than that in EOAD neurons (Figure 6D). Together, these results strongly suggest that LOAD neurons are more deficient in maintaining cellular redox homeostasis than EOAD neurons. Heme is an essential group in many antioxidant enzymes such as catalases and peroxidases, and is crucial for their functions. Thus, impaired heme homeostasis, as well as the known effects of APOE4 on redox balance 48, likely lead to elevated redox imbalance in these LOAD neurons.

### RNA-Seq analysis identifies diverse gene expression changes in AD neurons

We want to confirm the above findings indicating differences in cellular energy metabolism and redox homeostasis and to gain global insights into molecular characteristics of LOAD vs EOAD neurons. Therefore, we decided to carry out systematic RNA-Seq analysis. We performed the analysis using mRNAs isolated from undifferentiated and differentiated healthy control iPSC stem cells (healthy control), as well as undifferentiated and differentiated EOAD and LOAD iPSC stem cells. To identify potential alterations that underlie the initiation of LOAD vs EOAD, we wished to compare the changes between differentiated AD neurons vs differentiated control healthy neurons. However, iPSC stem cells from AD patients and healthy controls are far from isogenic, although they are matched in sex and APOE alleles to the extent possible. Thus, direct comparisons of AD vs non-isogenic controls may mask gene expression changes that impact AD etiopathogenesis. To mitigate this problem, we decided to identify gene expression changes induced by neuronal differentiation and compare these changes between AD and heathy controls. This is based on the reasoning that differentiated and undifferentiated cells of AD or healthy controls have identical genetic background, and their comparisons will identify gene expression changes that are most likely to be relevant to neuronal functions. Thus, the comparison of gene expression changes induced by neuronal differentiation between AD and healthy controls circumvents the issue of unknown genetic differences between AD and healthy control cells influencing gene expression patterns.

Thus, we identified genes whose expression was changed in differentiated vs undifferentiated iPSC cells from AD or healthy controls, as well as genes whose expression was changed in differentiated AD neurons vs differentiated control neurons. Briefly, genes with adjusted false discovery rate of less than 0.05 in given comparisons and with log_2_FoldChange of greater than 0.8 or less than -0.8 were selected as differentially expressed genes. Table 2 summarizes the numbers of differentially expressed genes in relevant comparisons. Notably, the numbers of common differentiation-induced gene expression changes between AD and healthy controls (Table 2; see numbers shared by HC M DIF/UD + PSEN1 DIF/UD and HC F DIF/UD + APOE4 DIF/UD) were as many as, or more than, the numbers of common differentiation-induced gene expression changes between EOAD and LOAD (Table 2; see numbers shared by PSEN1 DIF + APOE4 DIF) and between healthy controls (Table 2; see numbers shared by HC M DIF + HC F DIF). This indicates that genetic background differences between two healthy controls or between LOAD and EOAD can contribute as many differences as the APOE4 or PSEN1 mutation to gene expression changes induced by neuronal differentiation. Thus, comparison of genes altered in differentiated AD cells (vs undifferentiated) with genes altered in differentiated health control cells (vs undifferentiated) would circumvent the contributions from differing genetic background and have the potential to identify changes in gene expression caused by AD mutations and the difference between EOAD and LOAD.

### Analysis of gene expression patterns suggests that LOAD neurons, not EOAD neurons, are severely deficient in maintaining heme and redox homeostasis

**Figure 7:**
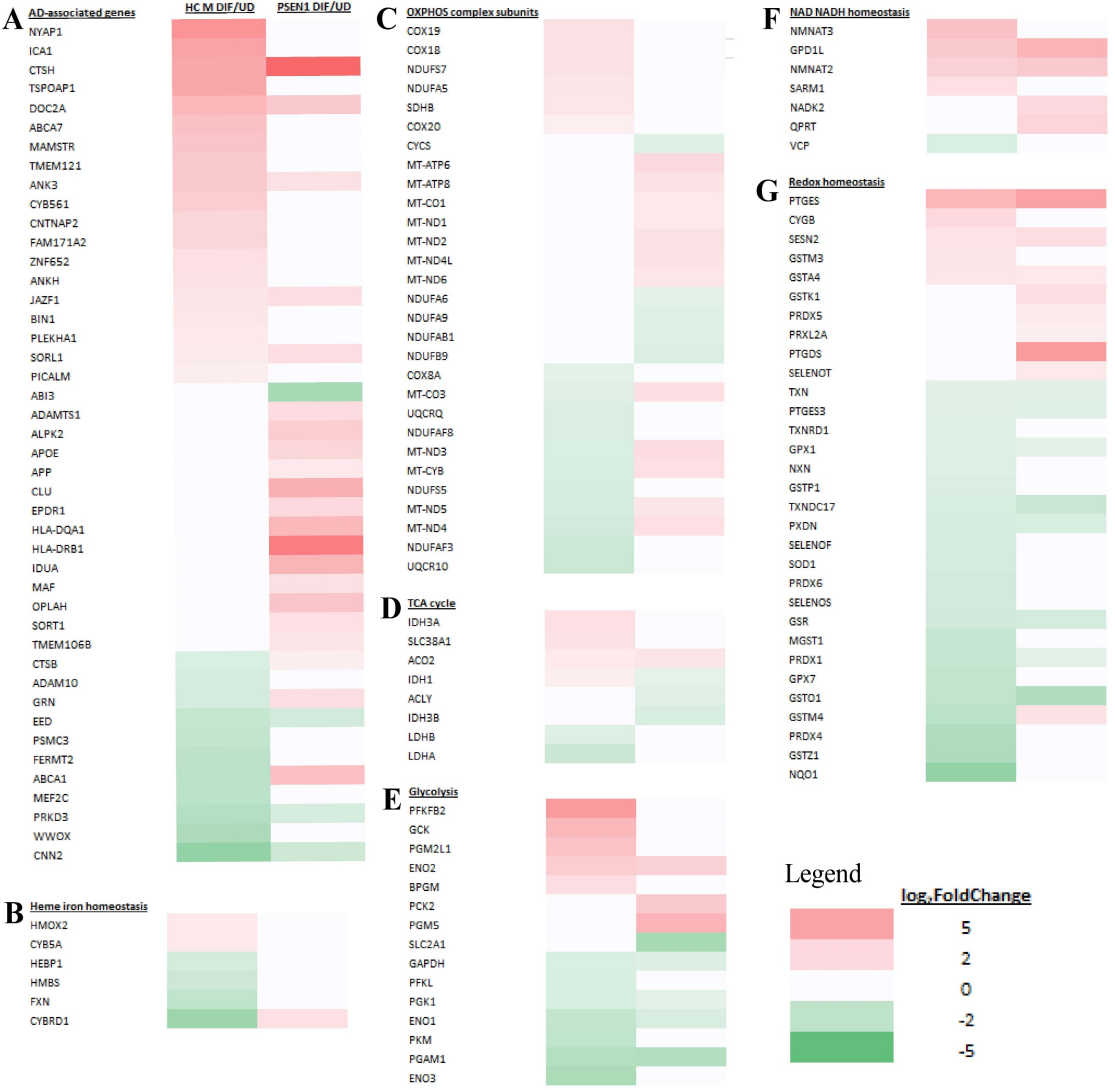
Heatmaps showing the patterns of transcript level changes induced by neuronal differentiation in EOAD neurons and heathy control (HC) neurons. Those known AD risk genes (A) and those genes relating to heme iron hemeostasis (B), OXPHOS complex subunits (C), TCA cycle (D), glycolysis (E), NAD NADH homeostasis (F), and redox homeostasis (G) whose expression was changed in differentiated EOAD (PSEN1) or its healthy control neurons (HC M) are shown.

**Figure 8:**
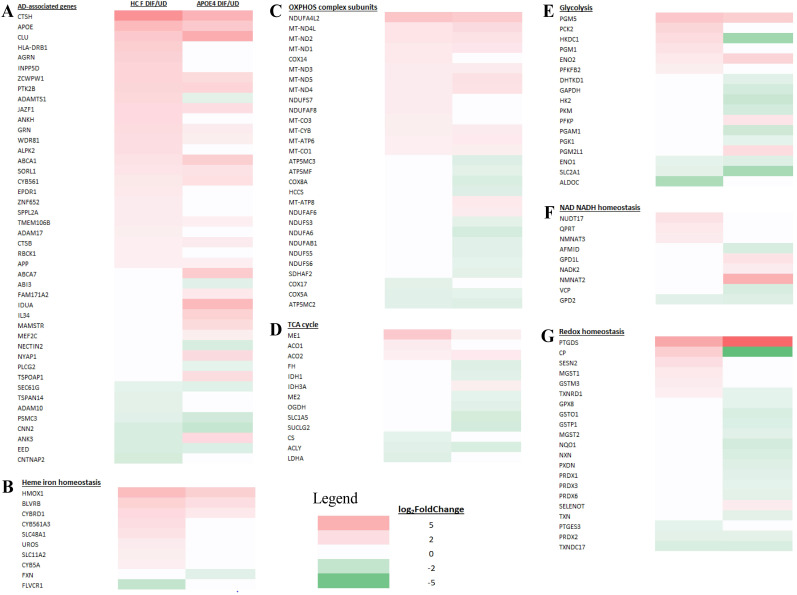
Heatmaps showing the patterns of transcript level changes induced by neuronal differentiation in LOAD neurons and heathy control (HC) neurons. Those known AD risk genes (A) and those genes relating to heme iron hemeostasis (B), OXPHOS complex subunits (C), TCA cycle (D), glycolysis (E), NAD NADH homeostasis (F), and redox homeostasis (G) whose expression was changed in differentiated LOAD (APOE4) or its healthy control neurons (HC F) are shown.

We examined and compared the genes whose expression is changed in differentiated AD neurons (compared to undifferentiated) and genes whose expression is changed in the corresponding healthy control neurons (compared to undifferentiated). Figure 7A-G show the transcript change patterns of those known AD-, cellular energy-, or redox-related genes whose expression was changed in differentiated EOAD (PSEN1) or its healthy control neurons (HC M). Figure 8A-G show the transcript change patterns of those known AD-, cellular energy-, or redox-related genes whose expression was changed in differentiated LOAD (APOE4) or its healthy control neurons (HC F). The details of these genes and transcript fold level changes are shown in Supplemental Tables S1 and S2.

First, we examined the expression patterns of over 100 known AD-associated genes identified via genome-wide association studies 24. Figure 7A and Supplemental Table S1 show that 44 AD-associated genes displayed altered expression in EOAD neurons or the healthy control neurons. Likewise, 44 AD-associated genes displayed altered expression in LOAD neurons or the healthy control neurons (Figure 8A and Supplemental Table S2). The altered expression of these genes during neuronal differentiation is consistent with their putative roles in neuronal functions and in AD etiopathogenesis.

Subsequently, we examined the expression patterns of genes associated with heme homeostasis, energy generation (OXPHOS, TCA cycle, and glycolysis), as well as NAD/NADH and redox homeostasis. We tried to identify expression changes that may disturb these crucial processes for neuronal functions. Particularly, reduced expression of many genes involved in heme, energy, and redox homeostasis is known to contribute neuronal dysfunction and presumably AD etiopathogenesis 31, 43, 49. We therefore identified genes whose expression is increased or unchanged in healthy control neurons (vs undifferentiated) but is increased much less, unchanged or decreased in AD neurons (vs undifferentiated). Such changes would indicate their expression, and by extension, their functions may be reduced in AD neurons compared to their corresponding healthy controls. Such reductions may contribute to the initiation and development of AD.

We examined how heme-related genes may be differentially affected in EOAD vs LOAD neurons. Data in Figure 7B and Supplemental Table S1 show that the expression of two heme-related genes (HMOX2 and CYB5A) was increased substantially in healthy neurons (vs undifferentiated) but was not changed in EOAD neurons by differentiation. Strikingly, the expression of 9 heme-related genes (HMOX1, BLVRB, CYBRD1, CYB561A3, SLC48A1, UROS, SLC11A2, CYB, and FXN, Figure 8B and Supplemental Table S2) was increased substantially in healthy neurons (vs undifferentiated) or not changed, but was less increased, not changed, or reduced in LOAD neurons by differentiation, respectively. This is also consistent with reduced levels of HO-1, HO-2, and BLVRB proteins shown in Figure 3. The diminished increase or reduction of transcript levels in many more genes involved in heme synthesis, uptake, and degradation in LOAD neurons indicate a greater deficiency in heme homeostasis in LOAD neurons compared to EOAD neurons. Note that transcript and protein levels of various genes do not always correlate. However, previous studies of heme-related gene expression have shown that the regulation of transcription, translation, and assembly of these enzymes/proteins is often coordinated and changes in the same up or down direction 50. Consequently, increases in proteins levels of heme and OXPHOS-related enzymes/proteins are often greater than those in transcript levels in cases such as cancer and AD 31, 48, 51. Additionally, the expression of heme exporter FLVCR1 was reduced in differentiated healthy control neurons but not changed in LOAD neurons (Figure 8B and Supplemental Table S2). This may exacerbate the decrease in intracellular heme pool caused by decreased heme synthesis in LOAD neurons relative to control neurons.

Examination of expression patterns of genes involved in OXPHOS, TCA cycle, and glycolysis showed a similar difference in the numbers of genes whose expression was changed by neuronal differentiation. Figure 7C-E and Supplemental Table S1 show that 11 genes relating to OXPHOS, 5 genes relating to TCA cycle, and 5 genes relating to glycolysis exhibited reduced extent of differentiation-induced expression changes, and likely neuronal functions, in EOAD neurons relative to healthy control neurons. Figure 8C-E and Supplemental Table S2 show that 14 genes relating to OXPHOS, 9 genes relating to TCA cycle, and 11 genes relating to glycolysis exhibited reduced extent of differentiation-induced expression changes, and likely neuronal function, in EOAD neurons relative to healthy control neurons.

Evidently, more genes involved in these bioenergetics' pathways exhibited diminished increase or reduction in LOAD neurons compared to EOAD neurons relative to their respective control neurons, although the differences in numbers are less dramatic as those involved in heme homeostasis. Nonetheless, the differences would indicate more severe deficiency of energy generation in LOAD neurons compared to EOAD neurons.

Next, we examined the changes in expression patterns of genes involved in NAD/NADH and redox homeostasis. Figure 7F & G and Supplemental Table S1 show that 2 genes involved in NAD/NADH metabolism and 2 genes involved in redox homeostasis exhibited reduced extent of differentiation-induced expression changes in EOAD neurons relative to healthy control neurons. In contrast, Figure 8F & G and Supplemental Table S2 show that 5 genes involved in NAD/NADH metabolism and 16 genes involved in redox homeostasis exhibited reduced extent of differentiation-induced expression changes in LOAD neurons relative to healthy control neurons. These results reveal a dramatic increase in the number of genes relating to redox homeostasis in LOAD, not EOAD, neurons whose expression was not maintained at levels similar to those in healthy control neurons. They suggest that LOAD neurons likely are more severely deficient in maintaining redox homeostasis, compared to EOAD neurons.

## Discussion

Although APOE4 is the strongest genetic risk factor for LOAD 10, 11, it is not the only factor causing AD pathogenesis in LOAD. APOE4 causes very different neurological outcomes in different patients 17-19. Thus, it is crucial to uncover those factors that may cooperate with APOE4 to cause LOAD. The AD-associated genes identified via genome-wide association studies have very small effect size in LOAD pathogenesis 24. Such genes may work together to impact certain molecular and cellular events to induce neural dysfunction. Thus, studies involving the use of CRISPR editing to generate isogenic cell lines with one mutant LOAD allele may not recapitulate the molecular changes in LOAD neurons. Here, we took advantage of two scarcely available pairs of human iPSC-derived neural stem cells, to uncover crucial molecular events that may initiate LOAD pathogenesis. Our use of two paired EOAD and LOAD human neurons affords a unique approach to identify *bona fide* molecular differences in LOAD vs EOAD neurons that are derived from genetic factors, not from aging or environmental factors. To our knowledge, this is the only study using two scarcely available pairs of EOAD and LOAD iPSC-derived neural stem cells with matched controls from healthy donors. Note that it is very difficult to find healthy matches for AD patient cells. Indeed, our study reveals novel insights into AD pathogenesis that were not uncovered by previous studies.

Because cellular rejuvenation linked to the reprograming of iPSC cells 25, 27, 28, our experiments using the iPSC-derived neurons may uncover only those cellular changes in AD vs control that are dictated by genetic changes in the somatic cells of donors, largely without the contribution from accumulated Aβ, tau, or environmental factors during the development of AD in humans and mouse models. Recently, advances in cancer immunology and autoimmune disease research have elicited a new wave of research in neuroimmunology 52. Both innate immunity involving microglia and adaptive immunity involving infections and T cells can cause neuroinflammation and result in neuronal dysfunction in neurodegenerative diseases including AD and Parkinson's disease (PD) 53. Altered levels and functions of T cells have been associated with AD pathology in numerous studies (for a review, see ref. 54). Recent studies have shown that microglia-mediated brain infiltration of T cells can mitigate cognitive decline or contribute to neurodegeneration in different mouse models of AD 55, 56. In neurodegenerative diseases, brain infiltration of T cells involves the presentation of either self-antigens that can be derived from aggregated proteins, such as Aβ and tau in AD or α-synuclein in PD, or non-self-antigens, such as those from viral or bacterial infections. In our study, iPSC-derived neurons were of fetal state and were not exposed to any antigens. Thus, it is not surprising that our study here did not identify any differences in neuroinflammatory factors in AD vs control neurons. This, however, does not necessarily diminish the potential significance of this study focusing on the comparison of neurons. Ultimately, neurodegeneration results from neuronal dysfunction. It is highly likely that altered neuronal functions linked to intrinsic genetic changes in AD patients contribute substantially to neurodegeneration, even though neuroinflammation may have a crucial role in neurodegeneration as well. Further, many questions regarding the interaction between the adaptive immune system and the CNS remain to be answered 52, 57. Thus, our study of cellular events in AD vs control neurons is still of high relevance to the understanding of AD etiopathogenesis.

**Figure 9:**
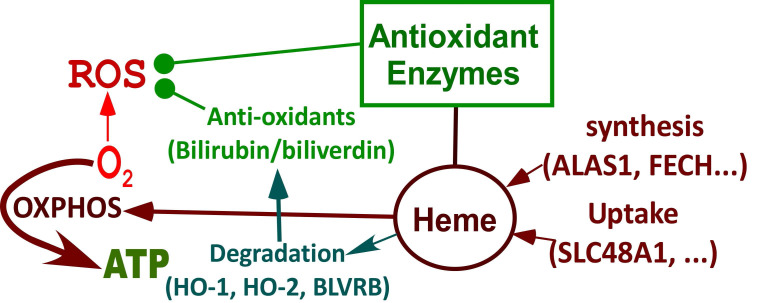
Summary of processes that are differentially affected in LOAD vs EOAD neurons. Highly induced pathways in neuronal differentiation but suppressed in LOAD neurons. During neuronal differentiation, heme synthesis and uptake are intensified to increase heme supply, which leads to increased production of OXPHOS complexes and oxygen consumption to generate energy. Heme degradation and the production of many antioxidant enzymes are also increased, which provide strong reducing power to counter ROS generated largely from OXPHOS. In LOAD neurons, the increases in heme synthesis, heme uptake, heme degradation, antioxidant enzymes, and OXPHOS are suppressed. This leads to deficiencies in heme homeostasis, redox homeostasis, and cellular energy metabolism, initiating neurological deficits associated with LOAD.

Our combined biochemical and RNA-Seq approaches to characterize differentiated and undifferentiated iPSC-derived neural stem cells from two pairs of LOAD-healthy and EOAD-healthy patients have yielded novel insights into the etiopathogenesis of LOAD vs EOAD. The results reveal that neurons from LOAD and EOAD patients possess distinct properties, particularly in heme and redox homeostasis. Our study identifies pathways that are defective in LOAD neurons, but not in EOAD neurons (see Figure 9). First, the availability of heme was substantially limited in LOAD neurons, but not EOAD neurons. Heme uptake was severely reduced in LOAD neurons, but not EOAD neurons (see Figure 4B). The changes in transcript levels of a heme transporter, SLC48A1, in EOAD and LOAD neurons are consistent with the above observation (Figures 7 & 8 and Supplemental Tables S1 & S2). This would lead to intracellular heme deficiency. Furthermore, the levels of heme synthesis enzyme FECH were increased in EOAD but decreased in LOAD neurons compared to their respective matched control neurons (Figure 3B). Likewise, the transcript level of another heme synthetic enzyme UROS was not changed in LOAD neurons, but increased in the control neurons (Figure 8 and Supplemental Table S2), indicating its decreased level and function in LOAD neurons relative to the matched control neurons. All these results strongly suggest that heme homeostasis is strongly perturbed in LOAD, not EOAD neurons. These multiple perturbations would severely limit the availability of heme in LOAD neurons.

Second, cell energy metabolism appeared to be more severely perturbed in LOAD neurons (relative to matched control neurons) than EOAD neurons (relative to matched control neurons). Figure 5A & B show that the levels of subunits of OXPHOS complexes COX5B and MTCO2 were increased or not changed in EOAD neurons (relative to matched control neurons), but were decreased in LOAD neurons (relative to matched control neurons). Consistent with this observation, RNA-Seq detected more genes involved in cellular energy generation whose transcript levels were reduced in LOAD neurons (accompanying differentiation; relative to matched control neurons) than in EOAD neurons (Figures 7 & 8 and Supplemental Tables S1 & S2). However, this difference between LOAD and EOAD is less dramatic compared to the difference in heme homeostasis described above or redox homeostasis discussed below.

Third, our data indicate that redox homeostasis was severely perturbed in LOAD neurons but not in EOAD neurons. Lipid oxidation detected by 4-HNE modification was not increased in EOAD neurons but increased in LOAD neurons (relative to control neurons, Figure 6C). Concurrently, ROS levels were not increased in EOAD neurons but were substantially increased in LOAD neurons (relative to matched control neurons, Figure 6D). Results from RNA-Seq analysis also indicated a dramatic perturbation of redox homeostasis in LOAD neurons, not in EOAD neurons. These results reveal that LOAD neurons, not EOAD neurons, exhibit severe, pre-existing defects in heme and redox homeostasis, as well as deficiencies in cell energy metabolism, including OXPHOS, TCA, and glycolysis.

The strongly perturbed redox homeostasis is likely attributable to multiple processes in LOAD neurons. As heme is a cofactor or prosthetic group in many antioxidant enzymes/proteins such as catalases and peroxidases, lowered intracellular heme levels would reduce the levels and activities of these enzymes. Additionally, multiple subunits of OXPHOS complexes require heme. Thus, lowered heme availability would also reduce their functions and enhance ROS generation. The presence of APOE4 in LOAD neurons also causes mitochondrial dysfunction and ROS generation 48. However, APOE4 per se does not cause such changes in heme and redox homeostasis as those we detected here. In a study using CRISPR/Cas9 and induced pluripotent stem cells (iPSCs) to examine APOE4 effects on human brain cells, Lin et al showed that the APOE4 variant can lead to extensive gene expression alterations in neurons, astrocytes, and microglia 58. However, they did not detect these dramatic changes in the heme- and redox-related genes that we detected here in LOAD neurons. These suggest that the changes in heme and redox homeostasis are not attributable to APOE4. Very likely, altered heme homeostasis is attributable to the interactions among the myriad non-APOE risk genes associated with LOAD. Reduced heme availability and APOE4 may act together to cause defective cellular energy generation and the buildup of ROS, initiating a cascade of events that impact the functions of other neural stem cells in the brain, ultimately leading to AD genesis.

It is well established that the risk of Alzheimer's disease associated with the *APOE* genotype is modulated by global and local genetic ancestries, other genetic risk loci and the lifetime exposome of an individual 10, 14, 59. Presumably, other environmental and genetic factors cooperate with APOE4 to cause the initial energy deficiency and redox imbalance in LOAD patients. As heme is such a crucial factor in both energy metabolism and redox homeostasis, it is not surprising that altered heme homeostasis can be a common factor in inducing neuronal dysfunction along with APOE4. It is plausible that heme and APOE can interact and influence each other. Indeed, heme uptake mediated by SLC48A1 (increased heme availability) can alleviate the effects of APOE knockout on oxLDL import 60. Likewise, brain endothelial cells expressing APOE4 have lowered levels of heme-related antioxidants 61. Thus, lower heme availability and APOE4 may act, directly or indirectly, to cooperatively disrupt neuronal functions and initiate AD pathogenesis. Indeed, APOE expression was strongly induced in LOAD neurons (Figure 8 and Supplemental Table S2). Further studies to illuminate the interactions of heme and APOE4 should deepen our understanding of the crucial neuronal events leading to the initiation and propagation of neural defects underlying AD pathology.

Taken together, these results strongly suggest that the etiopathogenesis of EOAD and LOAD involves distinct molecular and cellular mechanisms. We reason that the initiating molecular event of EOAD is the accumulation of Aβ as no substantial deficiencies in heme, energy, and redox homeostasis were observed in EOAD neurons. As Aβ accumulates, this would lead to AD pathology, as expected 62-64. In contrast, the initiating event of LOAD is likely pre-existing defects in heme and redox homeostasis, as well as perhaps in energy metabolism. Aβ has been shown to cause defects in heme, redox, and energy metabolism. Thus, these pre-existing defects would result in many neurological defects caused by Aβ accumulation 65, 66, which would initiate adverse effects on neuronal functions in LOAD patients. As these defects and the presence of APOE4 prolong, they will lead to the accumulation of Aβ 67, 68. Accumulation of Aβ will also cause further imbalanced heme and redox homeostasis 69, 70, which would further exacerbate the effects of heme and redox imbalance on neuronal functions. The accumulation of Aβ in LOAD neurons is also indicated by increased expression of APP protein (not shown) and transcript (Figure 8 and Supplemental Table S2) in LOAD and matched control neurons. These plaques will then cause a plethora of molecular and cellular changes in both EOAD and LOAD brains 71. Thus, despite the differences in molecular events promoting the initiation of neuronal defects in EOAD and LOAD, the AD pathology ultimately appears the same in both diseases.

Our results confirm and extend the recent finding that sporadic LOAD's development related to aging has distinct molecular features compared with inherited autosomal dominant EOAD 72. Our results suggest a novel hypothesis linking heme and redox homeostasis to LOAD that can positively impact AD research directions, although the lack of more available paired AD vs healthy control iPSC cell lines makes it necessary to further test this hypothesis using additional approaches. Nonetheless, this hypothesis does not conflict with any confirmed observations about AD. For example, the role of heme and redox homeostasis in LOAD does not exclude the roles of T cells in AD pathogenesis. Rather, altered heme and redox homeostasis are likely to contribute to dysregulation of T cell functions and cooperate with dysregulated immune responses in aged patients to cause AD pathogenesis. This hypothesis opens up new directions for investigating AD etiopathogenesis and therapeutic strategies. For example, it may be valuable to further investigate the contributions of genes relating to heme and redox homeostasis to AD etiopathogenesis. Additionally, the existing large-scale genomic, transcriptomic, proteomic, and metabolomics databases can be mined to uncover the roles of altered heme and redox homeostasis in AD pathogenesis. Our results suggest that the development of AD pathogenesis may be delayed or halted by rescuing heme and redox imbalance. This idea may guide future research to further evaluate the application of antioxidants in AD therapies. Clearly, the results reported here will be a novel addition to the repertoire of knowledge germane to Alzheimer's disease.

## Supplementary Material

Supplementary tables.

## Figures and Tables

**Table 1:** Information on cell lines used for healthy controls and for modeling AD phenotypes.

	AXOL Line	Age	Gender	Mutation	ApoE status
Control	ax0018	74	male		E2/E2
EOAD	ax0113	53	male	PSEN1 M146L	E2/E3
Control	ax0019	64	female		E3/E3
LOAD	ax0111	87	female		E4/E4

**Table 2:**
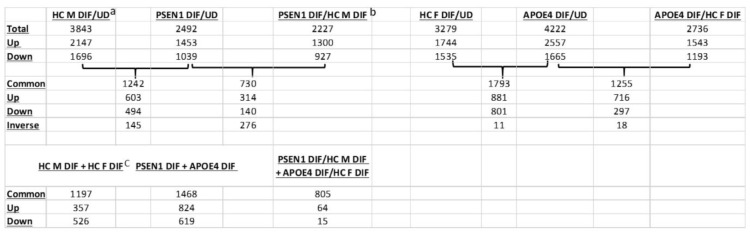
Comparisons of the number of genes altered in AD neurons and healthy control neurons in various combinations.

^a^The number of genes altered in the male healthy control neurons compared to undifferentiated iPSC cells. ^b^The number of common genes altered in AD PSEN1 neurons (compared to undifferentiated iPSC cells) shared with the healthy control neurons. ^c^The number of common genes altered in male health control neurons (compared undifferentiated iPSC cells) shared with the female healthy control neurons. HC: healthy control; PSEN1: cells with the PSEN1 L286V mutation; DIF: differentiated; UD: undifferentiated; Up: upregulated genes; Down: down regulated genes; Common: genes that are changed in both comparisons; Inverse: genes that are changed inversely in two comparisons.

## References

[1] Musiek ES, Holtzman DM (2015). Three dimensions of the amyloid hypothesis: time, space and 'wingmen'. Nat Neurosci.

[2] Montine TJ, Phelps CH, Beach TG, Bigio EH, Cairns NJ, Dickson DW (2012). National Institute on Aging-Alzheimer's Association guidelines for the neuropathologic assessment of Alzheimer's disease: a practical approach. Acta Neuropathol.

[3] Tellechea P, Pujol N, Esteve-Belloch P, Echeveste B, García-Eulate MR, Arbizu J (2018). Early- and late-onset Alzheimer disease: Are they the same entity?. Neurologia (Engl Ed).

[4] Mendez MF (2019). Early-onset Alzheimer Disease and Its Variants. Continuum (Minneap Minn).

[5] Bird TD (2008). Genetic aspects of Alzheimer disease. Genet Med.

[6] Cacace R, Sleegers K, Van Broeckhoven C (2016). Molecular genetics of early-onset Alzheimer's disease revisited. Alzheimer's & Dementia.

[7] Ayodele T, Rogaeva E, Kurup JT, Beecham G, Reitz C (2021). Early-Onset Alzheimer's Disease: What Is Missing in Research?. Curr Neurol Neurosci Rep.

[8] Hoogmartens J, Cacace R, Van Broeckhoven C (2021). Insight into the genetic etiology of Alzheimer's disease: A comprehensive review of the role of rare variants. Alzheimers Dement (Amst).

[9] Mielke MM (2018). Sex and Gender Differences in Alzheimer's Disease Dementia. Psychiatr Times.

[10] van der Lee SJ, Wolters FJ, Ikram MK, Hofman A, Ikram MA, Amin N (2018). The effect of APOE and other common genetic variants on the onset of Alzheimer's disease and dementia: a community-based cohort study. Lancet Neurol.

[11] Deming Y, Vasiljevic E, Morrow A, Miao J, Van Hulle C, Jonaitis E (2023). Neuropathology-based APOE genetic risk score better quantifies Alzheimer's risk. Alzheimers Dement.

[12] Huang Y, Mahley RW (2014). Apolipoprotein E: structure and function in lipid metabolism, neurobiology, and Alzheimer's diseases. Neurobiol Dis.

[13] Troutwine BR, Hamid L, Lysaker CR, Strope TA, Wilkins HM (2022). Apolipoprotein E and Alzheimer's disease. Acta Pharm Sin B.

[14] Jackson RJ, Hyman BT, Serrano-Pozo A (2024). Multifaceted roles of APOE in Alzheimer disease. Nature Reviews Neurology.

[15] Park KH, Noh Y, Choi EJ, Kim H, Chun S, Son YD (2017). Functional Connectivity of the Hippocampus in Early- and vs. Late-Onset Alzheimer's Disease. J Clin Neurol.

[16] Tort-Merino A, Falgàs N, Allen IE, Balasa M, Olives J, Contador J (2022). Early-onset Alzheimer's disease shows a distinct neuropsychological profile and more aggressive trajectories of cognitive decline than late-onset. Ann Clin Transl Neurol.

[17] Kim J, Woo SY, Kim S, Jang H, Kim J, Kim J (2021). Differential effects of risk factors on the cognitive trajectory of early- and late-onset Alzheimer's disease. Alzheimers Res Ther.

[18] Polsinelli AJ, Lane KA, Manchella MK, Logan PE, Gao S, Apostolova LG (2023). APOE ε4 is associated with earlier symptom onset in LOAD but later symptom onset in EOAD. Alzheimers Dement.

[19] Dong L, Li J, Liu C, Mao C, Wang J, Lei D (2021). Effects of ApoE genotype on clinical phenotypes in early-onset and late-onset Alzheimer's disease in China: Data from the PUMCH dementia cohort. Brain Behav.

[20] Beecham GW, Martin ER, Li Y-J, Slifer MA, Gilbert JR, Haines JL (2009). Genome-wide Association Study Implicates a Chromosome 12 Risk Locus for Late-Onset Alzheimer Disease. The American Journal of Human Genetics.

[21] Jonsson T, Atwal JK, Steinberg S, Snaedal J, Jonsson PV, Bjornsson S (2012). A mutation in APP protects against Alzheimer's disease and age-related cognitive decline. Nature.

[22] Wightman DP, Jansen IE, Savage JE, Shadrin AA, Bahrami S, Holland D (2021). A genome-wide association study with 1,126,563 individuals identifies new risk loci for Alzheimer's disease. Nature Genetics.

[23] Bellenguez C, Küçükali F, Jansen IE, Kleineidam L, Moreno-Grau S, Amin N (2022). New insights into the genetic etiology of Alzheimer's disease and related dementias. Nature Genetics.

[24] Andrews SJ, Renton AE, Fulton-Howard B, Podlesny-Drabiniok A, Marcora E, Goate AM (2023). The complex genetic architecture of Alzheimer's disease: novel insights and future directions. eBioMedicine.

[25] Studer L, Vera E, Cornacchia D (2015). Programming and Reprogramming Cellular Age in the Era of Induced Pluripotency. Cell Stem Cell.

[26] Mertens J, Reid D, Lau S, Kim Y, Gage FH (2018). Aging in a Dish: iPSC-Derived and Directly Induced Neurons for Studying Brain Aging and Age-Related Neurodegenerative Diseases. Annual Review of Genetics.

[27] Lapasset L, Milhavet O, Prieur A, Besnard E, Babled A, Aït-Hamou N (2011). Rejuvenating senescent and centenarian human cells by reprogramming through the pluripotent state. Genes & Development.

[28] Lee J, Bignone PA, Coles LS, Liu Y, Snyder E, Larocca D (2020). Induced pluripotency and spontaneous reversal of cellular aging in supercentenarian donor cells. Biochemical and Biophysical Research Communications.

[29] Aversano S, Caiazza C, Caiazzo M (2022). Induced pluripotent stem cell-derived and directly reprogrammed neurons to study neurodegenerative diseases: The impact of aging signatures. Frontiers in Aging Neuroscience. 2022; Volume 14 -.

[30] Kim HJ, Khalimonchuk O, Smith PM, Winge DR (2012). Structure, function, and assembly of heme centers in mitochondrial respiratory complexes. Biochim Biophys Acta.

[31] Vidal C, Daescu K, Fitzgerald KE, Starokadomska A, Bezprozvanny I, Zhang L (2019). Amyloid beta perturbs elevated heme flux induced with neuronal development. Alzheimers Dement (N Y).

[32] Chiabrando D, Fiorito V, Petrillo S, Tolosano E (2018). Unraveling the Role of Heme in Neurodegeneration. Front Neurosci.

[33] Wang Q, Tian J, Chen H, Du H, Guo L (2019). Amyloid beta-mediated KIF5A deficiency disrupts anterograde axonal mitochondrial movement. Neurobiol Dis.

[34] Marchenko S, Flanagan L Immunocytochemistry: human neural stem cells. J Vis Exp. 2007: 267.

[35] Sohoni S, Ghosh P, Wang T, Kalainayakan SP, Vidal C, Dey S (2019). Elevated Heme Synthesis and Uptake Underpin Intensified Oxidative Metabolism and Tumorigenic Functions in Non-Small Cell Lung Cancer Cells. Cancer Res.

[36] Dwyer BE, Smith MA, Richardson SL, Perry G, Zhu X (2009). Down-regulation of aminolevulinate synthase, the rate-limiting enzyme for heme biosynthesis in Alzheimer's disease. Neurosci Lett.

[37] Blumenfeld J, Yip O, Kim MJ, Huang Y (2024). Cell type-specific roles of APOE4 in Alzheimer disease. Nat Rev Neurosci.

[38] Vanova T, Sedmik J, Raska J, Amruz Cerna K, Taus P, Pospisilova V (2023). Cerebral organoids derived from patients with Alzheimer’s disease with PSEN1/2 mutations have defective tissue patterning and altered development. Cell Reports.

[39] Worthington MT, Cohn SM, Miller SK, Luo RQ, Berg CL (2001). Characterization of a human plasma membrane heme transporter in intestinal and hepatocyte cell lines. Am J Physiol Gastrointest Liver Physiol.

[40] Shayeghi M, Latunde-Dada GO, Oakhill JS, Laftah AH, Takeuchi K, Halliday N (2005). Identification of an intestinal heme transporter. Cell.

[41] Roy U, Yaish S, Weissman Z, Pinsky M, Dey S, Horev G (2022). Ferric reductase-related proteins mediate fungal heme acquisition. eLife.

[42] Lee H, Cho S, Kim MJ, Park YJ, Cho E, Jo YS (2023). ApoE4-dependent lysosomal cholesterol accumulation impairs mitochondrial homeostasis and oxidative phosphorylation in human astrocytes. Cell Rep.

[43] Orr AL, Kim C, Jimenez-Morales D, Newton BW, Johnson JR, Krogan NJ (2019). Neuronal Apolipoprotein E4 Expression Results in Proteome-Wide Alterations and Compromises Bioenergetic Capacity by Disrupting Mitochondrial Function. J Alzheimers Dis.

[44] Walker MA, Tian R (2018). NAD(H) in mitochondrial energy transduction: implications for health and disease. Curr Opin Physiol.

[45] Campbell JM (2022). Supplementation with NAD(+) and Its Precursors to Prevent Cognitive Decline across Disease Contexts. Nutrients.

[46] van der Velpen V, Rosenberg N, Maillard V, Teav T, Chatton JY, Gallart-Ayala H (2021). Sex-specific alterations in NAD+ metabolism in 3xTg Alzheimer's disease mouse brain assessed by quantitative targeted LC-MS. J Neurochem.

[47] Hou Y, Lautrup S, Cordonnier S, Wang Y, Croteau DL, Zavala E (2018). NAD(+) supplementation normalizes key Alzheimer's features and DNA damage responses in a new AD mouse model with introduced DNA repair deficiency. Proc Natl Acad Sci U S A.

[48] Mahley RW (2023). Apolipoprotein E4 targets mitochondria and the mitochondria-associated membrane complex in neuropathology, including Alzheimer's disease. Current Opinion in Neurobiology.

[49] Atamna H, Frey WH 2nd (2004). A role for heme in Alzheimer's disease: heme binds amyloid beta and has altered metabolism. Proc Natl Acad Sci U S A.

[50] Zhang L (2020). HEME BIOLOGY: Heme Acts as a Versatile Signaling Molecule Regulating Diverse Biological Processes Second ed. Singapore: World Scientific Publishing Company.

[51] Hooda J, Cadinu D, Alam MM, Shah A, Cao TM, Sullivan LA (2013). Enhanced heme function and mitochondrial respiration promote the progression of lung cancer cells. PLoS One.

[52] Elyaman W, Stern LJ, Jiang N, Dressman D, Bradley P, Klatzmann D (2025). Exploring the role of T cells in Alzheimer's and other neurodegenerative diseases: Emerging therapeutic insights from the T Cells in the Brain symposium. Alzheimer's & Dementia.

[53] Machhi J, Kevadiya BD, Muhammad IK, Herskovitz J, Olson KE, Mosley RL (2020). Harnessing regulatory T cell neuroprotective activities for treatment of neurodegenerative disorders. Mol Neurodegener.

[54] Afsar A, Chen M, Xuan Z, Zhang L (2023). A glance through the effects of CD4(+) T cells, CD8(+) T cells, and cytokines on Alzheimer's disease. Comput Struct Biotechnol J.

[55] Chen X, Firulyova M, Manis M, Herz J, Smirnov I, Aladyeva E (2023). Microglia-mediated T cell infiltration drives neurodegeneration in tauopathy. Nature.

[56] Su W, Saravia J, Risch I, Rankin S, Guy C, Chapman NM (2023). CXCR6 orchestrates brain CD8(+) T cell residency and limits mouse Alzheimer's disease pathology. Nat Immunol.

[57] Boskovic P, Gao W, Kipnis J (2024). Will cellular immunotherapies end neurodegenerative diseases?. Trends in Immunology.

[58] Lin YT, Seo J, Gao F, Feldman HM, Wen HL, Penney J (2018). APOE4 Causes Widespread Molecular and Cellular Alterations Associated with Alzheimer's Disease Phenotypes in Human iPSC-Derived Brain Cell Types. Neuron.

[59] Belloy ME, Andrews SJ, Le Guen Y, Cuccaro M, Farrer LA, Napolioni V (2023). APOE Genotype and Alzheimer Disease Risk Across Age, Sex, and Population Ancestry. JAMA Neurol.

[60] Pham TT, Le AH, Dang CP, Chong SY, Do DV, Peng B (2023). Endocytosis of red blood cell extracellular vesicles by macrophages leads to cytoplasmic heme release and prevents foam cell formation in atherosclerosis. J Extracell Vesicles.

[61] Marottoli FM, Trevino TN, Geng X, Arbieva Z, Kanabar P, Maienschein-Cline M (2021). Autocrine Effects of Brain Endothelial Cell-Produced Human Apolipoprotein E on Metabolism and Inflammation in vitro. Front Cell Dev Biol.

[62] Ricciarelli R, Fedele E (2017). The Amyloid Cascade Hypothesis in Alzheimer's Disease: It's Time to Change Our Mind. Curr Neuropharmacol.

[63] Zhang Y, Chen H, Li R, Sterling K, Song W (2023). Amyloid β-based therapy for Alzheimer's disease: challenges, successes and future. Signal Transduction and Targeted Therapy.

[64] Hampel H, Hardy J, Blennow K, Chen C, Perry G, Kim SH (2021). The Amyloid-β Pathway in Alzheimer's Disease. Molecular Psychiatry.

[65] Afsar A, Chacon Castro MdC, Soladogun AS, Zhang L (2023). Recent Development in the Understanding of Molecular and Cellular Mechanisms Underlying the Etiopathogenesis of Alzheimer's Disease. International Journal of Molecular Sciences.

[66] Afsar A, Zhang L (2024). Putative Molecular Mechanisms Underpinning the Inverse Roles of Mitochondrial Respiration and Heme Function in Lung Cancer and Alzheimer's Disease. Biology (Basel).

[67] Yamazaki Y, Zhao N, Caulfield TR, Liu C-C, Bu G (2019). Apolipoprotein E and Alzheimer disease: pathobiology and targeting strategies. Nature Reviews Neurology.

[68] Hunsberger HC, Pinky PD, Smith W, Suppiramaniam V, Reed MN (2019). The role of APOE4 in Alzheimer's disease: strategies for future therapeutic interventions. Neuronal Signal.

[69] Ghosh C, Seal M, Mukherjee S, Ghosh Dey S (2015). Alzheimer's Disease: A Heme-Aβ Perspective. Accounts of Chemical Research.

[70] Butterfield DA, Boyd-Kimball D (2019). Redox proteomics and amyloid β-peptide: insights into Alzheimer disease. J Neurochem.

[71] Zhang H, Li X, Wang X, Xu J, Elefant F, Wang J (2023). Cellular response to β-amyloid neurotoxicity in Alzheimer's disease and implications in new therapeutics. Animal Model Exp Med.

[72] Sun Z, Kwon JS, Ren Y, Chen S, Walker CK, Lu X (2024). Modeling late-onset Alzheimer's disease neuropathology via direct neuronal reprogramming. Science.

