# Supplementary material for: Altered Heme and Redox Homeostasis Underpin Late-onset Alzheimer's Disease
Source: Int J Biol Sci. 2025 Aug 22;21(12):5393–410. doi: 10.7150/ijbs.116204 (PMC12435994; doi:10.7150/ijbs.116204)
Supplement: Supplementary file 1 — Supplementary tables. [file ijbsv21p5393s1.pdf]

Supplemental Materials

Table S1 Comparison of transcript level changes of selected classes of genes induced by neuronal differentiation in EOAD (PSEN1) vs matched healthy control (HC)

Gene Name	Description	HC M DIF/UD	PSEN1 DIF/UD
	AD-associated genes	log_2FoldChange	log_2FoldChange
NYAP1	neuronal tyrosine phosphoryla phosphoinositide-3-kinase adaptor 1	5.8	nc
ICA1	islet cell autoantigen 1	4.9	nc
CTSH	cathepsin H	4.8	8.2
TSPOAP1	TSPO associated protein 1	4.8	nc
DOC2A	double C2 domain alpha	3.9	2.8
ABCA7	ATP binding cassette subfamily A member 7	3.3	nc
MAMSTR	MEF2 activating motif and SAP domain containing transcriptional regulator	3.0	nc
TMEM121	transmembrane protein 121	2.8	nc
ANK3	ankyrin 3	2.8	1.5
CYB561	cytochrome b561	2.6	nc
CNTNAP2	contactin associated protein like 2	2.2	nc
FAM171A2	family with sequence similarity 171 member A2	2.1	nc
ZNF652	zinc finger protein 652	1.6	nc
ANKH	ANKH inorganic pyrophosphate transport regulator	1.5	nc
JAZF1	JAZF zinc finger 1	1.3	1.6
BIN1	bridging integrator 1	1.3	nc
PLEKHA1	pleckstrin homology domain containing A1	1.1	nc
SORL1	sortilin related receptor 1	1.0	1.7
PICALM	phosphatidylinositol binding clathrin assembly protein	0.8	nc
ABI3	ABI family member 3	nc	-2.8
ADAMTS1	ADAM metallopeptidase with thrombospondin type 1 motif 1	nc	1.8
ALPK2	alpha kinase 2	nc	2.6

APOE	apolipoprotein E	nc	2.1
APP	amyloid beta precursor protein	nc	1.2
CLU	clusterin	nc	4.3
EPDR1	ependymin related 1	nc	1.9
HLA-DQA1	major histocompatibility complex, class II, DQ alpha 1	nc	3.8
HLA-DRB1	major histocompatibility complex, class II, DR beta 1	nc	7.0
IDUA	iduronidase, alpha-L-	nc	4.0
MAF	MAF bZIP transcription factor	nc	1.5
OPLAH	5-oxoprolinase, ATP-hydrolysing	nc	3.0
SORT1	sortilin 1	nc	1.6
TMEM106B	transmembrane protein 106B	nc	1.3
CTSB	cathepsin B	-1.0	0.9
ADAM10	ADAM metallopeptidase domain 10	-1.2	nc
GRN	granulin precursor	-1.3	1.7
EED	embryonic ectoderm development	-1.7	-1.3
PSMC3	proteasome 26S subunit, ATPase 3	-1.9	nc
FERMT2	fermitin family member 2	-2.0	nc
ABCA1	ATP binding cassette subfamily A member 1	-2.1	3.3
MEF2C	myocyte enhancer factor 2C	-2.1	nc
PRKD3	protein kinase D3	-2.3	-1.2
WWOX	WW domain containing oxidoreductase	-2.5	nc
CNN2	calponin 2	-3.3	-1.5
	**Heme iron homeostasis**	-	-
HMOX2	heme oxygenase 2	1.2*	nc
CYB5A	cytochrome b5 type A	1.2	nc
HEBP1	heme binding protein 1	-1.3	nc
HMBS	hydroxymethylbilane synthase	-1.6	nc
FXN	frataxin	-1.9	nc
CYBRD1	cytochrome b reductase 1	-3.1	1.6
	**OXPHOS complex subunits**		
COX19	cytochrome c oxidase assembly factor COX19	1.5	nc

COX18	cytochrome c oxidase assembly factor COX18	1.5	nc
NDUFS7	NADH:ubiquinone oxidoreductase core subunit S7	1.4	nc
NDUFA5	NADH:ubiquinone oxidoreductase subunit A5	1.3	nc
SDHB	succinate dehydrogenase complex iron sulfur subunit B	1.2	nc
COX20	cytochrome c oxidase assembly factor COX20	0.8	nc
CYCS	cytochrome c, somatic	nc	-0.9
MT-ATP6	mitochondrially encoded ATP synthase membrane subunit 6	nc	1.9
MT-ATP8	mitochondrially encoded ATP synthase membrane subunit 8	nc	1.5
MT-CO1	mitochondrially encoded cytochrome c oxidase I	nc	1.2
MT-ND1	mitochondrially encoded NADH:ubiquinone oxidoreductase core subunit 1	nc	1.1
MT-ND2	mitochondrially encoded NADH:ubiquinone oxidoreductase core subunit 2	nc	1.5
MT-ND4L	mitochondrially encoded NADH:ubiquinone oxidoreductase core subunit 4L	nc	1.5
MT-ND6	mitochondrially encoded NADH:ubiquinone oxidoreductase core subunit 6	nc	1.2
NDUFA6	NADH:ubiquinone oxidoreductase subunit A6	nc	-0.8
NDUFA9	NADH:ubiquinone oxidoreductase subunit A9	nc	-1.0
NDUFAB1	NADH:ubiquinone oxidoreductase subunit AB1	nc	-0.9
NDUFB9	NADH:ubiquinone oxidoreductase subunit B9	nc	-1.0
COX8A	cytochrome c oxidase subunit 8A	-0.8	nc
MT-CO3	mitochondrially encoded cytochrome c oxidase III	-0.9	1.6
UQCRQ	ubiquinol-cytochrome c reductase complex III subunit VII	-1.0	nc
NDUFAF8	NADH:ubiquinone oxidoreductase	-1.0	nc

	complex assembly factor 8		
MT-ND3	mitochondrially encoded NADH:ubiquinone oxidoreductase core subunit 3	-1.0	1.8
MT-CYB	mitochondrially encoded cytochrome b	-1.1	1.6
NDUFS5	NADH:ubiquinone oxidoreductase subunit S5	-1.2	nc
MT-ND5	mitochondrially encoded NADH:ubiquinone oxidoreductase core subunit 5	-1.3	1.3
MT-ND4	mitochondrially encoded NADH:ubiquinone oxidoreductase core subunit 4	-1.4	1.6
NDUFAF3	NADH:ubiquinone oxidoreductase complex assembly factor 3	-1.6	nc
UQCR10	ubiquinol-cytochrome c reductase, complex III subunit X	-1.6	nc
	**TCA cycle**		
IDH3A	isocitrate dehydrogenase 3 (NAD(+)) alpha	1.6	nc
SLC38A1	solute carrier family 38 member 1 (glutamine)	1.4	nc
ACO2	aconitase 2	1.0	1.3
IDH1	isocitrate dehydrogenase (NADP(+)) 1, cytosolic	0.9	-0.8
ACLY	ATP citrate lyase	nc	-0.9
IDH3B	isocitrate dehydrogenase 3 (NAD(+)) beta	nc	-1.1
LDHB	lactate dehydrogenase B	-1.0	nc
LDHA	lactate dehydrogenase A	-1.6	nc
	**Glycolysis**		
PFKFB2	6-phosphofructo-2-kinase/fructose-2,6-biphosphatase 2	5.5	nc
GCK	glucokinase	3.8	nc
PGM2L1	phosphoglucomutase 2 like 1	3.2	nc
ENO2	enolase 2	2.6	2.3
BPGM	bisphosphoglycerate mutase	1.8	nc
PCK2	phosphoenolpyruvate carboxykinase 2, mitochondrial	nc	2.9

PGM5	phosphoglucomutase 5	nc	4.1
SLC2A1	solute carrier family 2 member 1 (Glut 1)	nc	-2.7
GAPDH	glyceraldehyde-3-phosphate dehydrogenase	-1.0	-1.0
PFKL	phosphofructokinase, liver type	-1.1	nc
PGK1	phosphoglycerate kinase 1	-1.1	-0.8
ENO1	enolase 1	-1.8	-1.1
PKM	pyruvate kinase M1/2	-1.9	nc
PGAM1	phosphoglycerate mutase 1	-2.3	-2.4
ENO3	enolase 3	-2.6	nc
	**NAD NADH homeostasis**		
NMNAT3	nicotinamide nucleotide adenylyltransferase 3	3.3	nc
GPD1L	glycerol-3-phosphate dehydrogenase 1 like	2.9	4.1
NMNAT2	nicotinamide nucleotide adenylyltransferase 2	2.3	2.9
SARM1	sterile alpha and TIR motif containing 1	1.6	nc
NADK2	NAD kinase 2, mitochondrial	nc	1.9
QPRT	quinolinate phosphoribosyltransferase	nc	2.2
VCP	valosin containing protein	-1.0	nc
-	**Redox homeostasis**	-	-
PTGES	prostaglandin E synthase	3.9	5.1
CYGB	cytoglobin	1.9	nc
SESN2	sestrin 2	1.4	1.8
GSTM3	glutathione S-transferase mu 3	1.3	nc
GSTA4	glutathione S-transferase alpha 4	1.2	1.1
GSTK1	glutathione S-transferase kappa 1	nc	1.6
PRDX5	peroxiredoxin 5	nc	1.0
PRXL2A	peroxiredoxin like 2A	nc	0.9
PTGDS	prostaglandin D2 synthase	nc	5.4
SELENOT	selenoprotein T	nc	1.2
TXN	thioredoxin	-0.9	-0.9
PTGES3	prostaglandin E synthase 3	-0.9	-0.9

TXNRD1	thioredoxin reductase 1	-0.9	nc
GPX1	glutathione peroxidase 1	-0.9	-0.8
NXN	nucleoredoxin	-1.0	nc
GSTP1	glutathione S-transferase pi 1	-1.0	nc
TXNDC17	thioredoxin domain containing 17	-1.1	-1.7
PXDN	peroxidasin	-1.2	-1.1
SELENOF	selenoprotein F	-1.2	nc
SOD1	superoxide dismutase 1	-1.3	nc
PRDX6	peroxiredoxin 6	-1.3	nc
SELENOS	selenoprotein S	-1.4	nc
GSR	glutathione-disulfide reductase	-1.4	-1.3
MGST1	microsomal glutathione S-transferase 1	-1.7	nc
PRDX1	peroxiredoxin 1	-1.7	-0.8
GPX7	glutathione peroxidase 7	-1.8	nc
GSTO1	glutathione S-transferase omega 1	-1.9	-2.5
GSTM4	glutathione S-transferase mu 4	-2.1	1.5
PRDX4	peroxiredoxin 4	-2.4	nc
GSTZ1	glutathione S-transferase zeta 1	-2.5	nc
NQO1	NAD(P)H quinone dehydrogenase 1	-3.3	nc

*Underline indicates the changes in EOAD vs WT were substantially different in direction or in degree.

Table S2 Comparison of transcript level changes of selected classes of genes induced by neuronal differentiation in LOAD (APOE4) vs matched healthy control (HC)

Gene Name	Description	HC F DIF/UD	APOE4 DIF/UD
	AD-associated genes	log_2FoldChange	log_2FoldChange
CTSH	cathepsin H	6.7	4.6
APOE	apolipoprotein E	4.3	3.3
CLU	clusterin	3.4	5.2
HLA-DRB1	major histocompatibility complex, class II, DR beta 1	2.9	nc
AGRN	agrin	2.7	nc
INPP5D	inositol polyphosphate-5-phosphatase D	2.5	nc

ZCWPW1	zinc finger CW-type and PWWP domain containing 1	2.5	2.2
PTK2B	protein tyrosine kinase 2 beta	2.4	2.6
ADAMTS1	ADAM metallopeptidase with thrombospondin type 1 motif 1	2.3	-0.9
JAZF1	JAZF zinc finger 1	2.3	1.9
ANKH	ANKH inorganic pyrophosphate transport regulator	2.2	nc
GRN	granulin precursor	2.1	1.1
WDR81	WD repeat domain 81	2.0	1.0
ALPK2	alpha kinase 2	2.0	nc
ABCA1	ATP binding cassette subfamily A member 1	1.6	3.0
SORL1	sortilin related receptor 1	1.5	1.6
CYB561	cytochrome b561	1.4	1.8
EPDR1	ependymin related 1	1.4	nc
ZNF652	zinc finger protein 652	1.1	nc
SPPL2A	signal peptide peptidase like 2A	1.0	nc
TMEM106B	transmembrane protein 106B	1.0	0.9
ADAM17	ADAM metallopeptidase domain 17	1.0	nc
CTSB	cathepsin B	0.9	1.1
RBCK1	RANBP2-type and C3HC4-type zinc finger containing 1	0.9	nc
APP	amyloid beta precursor protein	0.8	0.8
ABCA7	ATP binding cassette subfamily A member 7	nc	3.2
ABI3	ABI family member 3	nc	-0.9
FAM171A2	family with sequence similarity 171 member A2	nc	1.3
IDUA	iduronidase, alpha-L-	nc	4.2
IL34	interleukin 34	nc	2.8
MAMSTR	MEF2 activating motif and SAP domain containing transcriptional regulator	nc	2.3
MEF2C	myocyte enhancer factor 2C	nc	1.0
NECTIN2	nectin cell adhesion molecule 2	nc	-1.3
NYAP1	neuronal tyrosine phosphorylated phosphoinositide-3-kinase adaptor 1	nc	2.2
PLCG2	phospholipase C gamma 2	nc	-0.8
TSPOAP1	TSPO associated protein 1	nc	2.0
SEC61G	Sec61 translocon gamma subunit	-0.8	-1.0

TSPAN14	tetraspanin 14	-0.9	nc
ADAM10	ADAM metallopeptidase domain 10	-0.9	nc
PSMC3	proteasome 26S subunit, ATPase 3	-1.0	-1.6
CNN2	calponin 2	-1.3	-1.9
ANK3	ankyrin 3	-1.3	2.2
EED	embryonic ectoderm development	-1.4	-1.2
CNTNAP2	contactin associated protein like 2	-1.4	nc
	**Heme iron homeostasis**		
HMOX1	heme oxygenase 1	4.1*	2.9
BLVRB	biliverdin reductase B	2.8	2.0
CYBRD1	cytochrome b reductase 1	2.2	1.3
CYB561A3	cytochrome b561 family member A3	2.0	nc
SLC48A1	solute carrier family 48 member 1	1.6	nc
UROS	uroporphyrinogen III synthase	1.1	nc
SLC11A2	solute carrier family 11 member 2	1.0	nc
CYB5A	cytochrome b5 type A	0.9	nc
FXN	frataxin	nc	-1.0
FLVCR1	feline leukemia virus subgroup C cellular receptor 1	-2.1	nc
	**OXPHOS complex subunits**		
NDUFA4L2	NDUFA4, mitochondrial complex associated like 2	3.5	3.2
MT-ND4L	mitochondrially encoded NADH:ubiquinone oxidoreductase core subunit 4L	1.6	2.1
MT-ND2	mitochondrially encoded NADH:ubiquinone oxidoreductase core subunit 2	1.6	1.7
MT-ND1	mitochondrially encoded NADH:ubiquinone oxidoreductase core subunit 1	1.4	1.5
COX14	cytochrome c oxidase assembly factor COX14	1.3	nc
MT-ND3	mitochondrially encoded NADH:ubiquinone oxidoreductase core subunit 3	1.1	1.2
MT-ND5	mitochondrially encoded NADH:ubiquinone oxidoreductase core subunit 5	1.1	1.8
MT-ND4	mitochondrially encoded NADH:ubiquinone oxidoreductase core subunit 4	1.1	1.7
NDUFS7	NADH:ubiquinone oxidoreductase core subunit S7	1.1	nc

NDUFAF8	NADH:ubiquinone oxidoreductase complex assembly factor 8	<u>1.1</u>	<u>nc</u>
MT-CO3	mitochondrially encoded cytochrome c oxidase III	<u>1.0</u>	<u>nc</u>
MT-CYB	mitochondrially encoded cytochrome b	1.0	1.1
MT-ATP6	mitochondrially encoded ATP synthase membrane subunit 6	0.9	1.2
MT-CO1	mitochondrially encoded cytochrome c oxidase I	0.9	1.0
ATP5MC3	ATP synthase membrane subunit c locus 3	<u>nc</u>	<u>-1.1</u>
ATP5MF	ATP synthase membrane subunit f	<u>nc</u>	<u>-0.9</u>
COX8A	cytochrome c oxidase subunit 8A	<u>nc</u>	<u>-1.3</u>
HCCS	holocytochrome c synthase	<u>nc</u>	<u>-1.1</u>
MT-ATP8	mitochondrially encoded ATP synthase membrane subunit 8	<u>nc</u>	1.3
NDUFAF6	NADH:ubiquinone oxidoreductase complex assembly factor 6	<u>nc</u>	1.1
NDUFS3	NADH:ubiquinone oxidoreductase core subunit S3	<u>nc</u>	<u>-0.9</u>
NDUFA6	NADH:ubiquinone oxidoreductase subunit A6	<u>nc</u>	<u>-1.5</u>
NDUFAB1	NADH:ubiquinone oxidoreductase subunit AB1	<u>nc</u>	<u>-1.0</u>
NDUFS5	NADH:ubiquinone oxidoreductase subunit S5	<u>nc</u>	<u>-1.0</u>
NDUFS6	NADH:ubiquinone oxidoreductase subunit S6	<u>nc</u>	<u>-0.8</u>
SDHAF2	succinate dehydrogenase complex assembly factor 2	<u>nc</u>	<u>-0.9</u>
COX17	cytochrome c oxidase copper chaperone COX17	-0.9	nc
COX5A	cytochrome c oxidase subunit 5A	-0.9	-0.9
ATP5MC2	ATP synthase membrane subunit c locus 2	-1.0	-1.1
	**<u>TCA cycle</u>**		
ME1	malic enzyme 1	<u>3.3</u>	<u>1.0</u>
ACO1	aconitase 1	<u>1.0</u>	<u>nc</u>
ACO2	aconitase 2	0.9	1.2
FH	fumarate hydratase	<u>nc</u>	<u>-1.1</u>
IDH1	isocitrate dehydrogenase (NADP(+)) 1, cytosolic	<u>nc</u>	<u>-1.0</u>
IDH3A	isocitrate dehydrogenase 3 (NAD(+)) alpha	<u>nc</u>	<u>1.0</u>
ME2	malic enzyme 2	<u>nc</u>	<u>-0.8</u>

OGDH	oxoglutarate dehydrogenase	nc	-1.0
SLC1A5	solute carrier family 1 member 5 (glutamine)	nc	-1.4
SUCLG2	succinate-CoA ligase GDP-forming beta subunit	nc	-1.5
CS	citrate synthase	-0.9	nc
ACLY	ATP citrate lyase	-1.0	-1.2
LDHA	lactate dehydrogenase A	-1.1	nc
	**Glycolysis**		
PGM5	phosphoglucomutase 5	3.4	2.9
PCK2	phosphoenolpyruvate carboxykinase 2, mitochondrial	2.4	nc
HKDC1	hexokinase domain containing 1	2.1	-3.4
PGM1	phosphoglucomutase 1	1.6	nc
ENO2	enolase 2	1.3	2.6
PFKFB2	6-phosphofructo-2-kinase/fructose-2,6-biphosphatase 2	1.0	nc
DHTKD1	dehydrogenase E1 and transketolase domain containing 1	nc	-1.0
GAPDH	glyceraldehyde-3-phosphate dehydrogenase	nc	-1.5
HK2	hexokinase 2	nc	-1.8
PKM	pyruvate kinase M1/2	nc	-1.5
PFKP	phosphofructokinase, platelet	nc	1.5
PGAM1	phosphoglycerate mutase 1	nc	-1.7
PGK1	phosphoglycerate kinase 1	nc	-0.9
PGM2L1	phosphoglucomutase 2 like 1	nc	2.1
ENO1	enolase 1	-0.8	-1.1
SLC2A1	solute carrier family 2 member 1 (Glut 1)	-0.9	-3.0
ALDOC	aldolase, fructose-bisphosphate C	-2.9	nc
	**NAD NADH homeostasis**		
NUDT17	nudix hydrolase 17	1.8	nc
QPRT	quinolinate phosphoribosyltransferase	1.3	nc
NMNAT3	nicotinamide nucleotide adenylyltransferase 3	1.1	nc
AFMID	arylformamidase	nc	-1.3
GPD1L	glycerol-3-phosphate dehydrogenase 1 like	nc	1.6
NADK2	NAD kinase 2, mitochondrial	nc	1.0

NMNAT2	nicotinamide nucleotide adenylyltransferase 2	nc	4.9
VCP	valosin containing protein	nc	-1.3
GPD2	glycerol-3-phosphate dehydrogenase 2	-1.0	-1.1
-	**Redox homeostasis**		
PTGDS	prostaglandin D2 synthase	5.5	9.4
CP	ceruloplasmin	3.0	-5.6
SESN2	sestrin 2	2.0	nc
MGST1	microsomal glutathione S-transferase 1	1.3	nc
GSTM3	glutathione S-transferase mu 3	1.0	nc
TXNRD1	thioredoxin reductase 1	0.8	-0.8
GPX8	glutathione peroxidase 8 (putative)	nc	-0.8
GSTO1	glutathione S-transferase omega 1	nc	-1.2
GSTP1	glutathione S-transferase pi 1	nc	-1.2
MGST2	microsomal glutathione S-transferase 2	nc	-1.0
NQO1	NAD(P)H quinone dehydrogenase 1	nc	-1.6
NXN	nucleoredoxin	nc	-1.2
PXDN	peroxidasin	nc	-1.1
PRDX1	peroxiredoxin 1	nc	-0.9
PRDX3	peroxiredoxin 3	nc	-0.8
PRDX6	peroxiredoxin 6	nc	-0.9
SELENOT	selenoprotein T	nc	1.1
TXN	thioredoxin	nc	-0.9
PTGES3	prostaglandin E synthase 3	-0.8	nc
PRDX2	peroxiredoxin 2	-0.9	-0.9
TXNDC17	thioredoxin domain containing 17	-1.2	-1.2

*Underline indicates the changes in EOAD vs WT were substantially different in direction or in degree.
